# The *Bifidobacterium*-dominated fecal microbiome in dairy calves shapes the characteristic growth phenotype of host

**DOI:** 10.1038/s41522-024-00534-4

**Published:** 2024-07-21

**Authors:** Yimin Zhuang, Shuai Liu, Duo Gao, Yiming Xu, Wen Jiang, Tianyu Chen, Jianxin Xiao, Jingjun Wang, Guobin Hou, Sumin Li, Xinjie Zhao, Yanting Huang, Shangru Li, Siyuan Zhang, Mengmeng Li, Wei Wang, Shengli Li, Zhijun Cao

**Affiliations:** 1 State Key Laboratory of Animal Nutrition and Feeding, College of Animal Science and Technology, China Agricultural University, Beijing, China; 2 College of Animal Science, Xinjiang Agricultural University, Urumqi, Xinjiang Uygur Autonomous Region China; 3 Animal Nutrition Institute, Sichuan Agricultural University, Chengdu, China; 4 College of Animal Science and Technology, Beijing University of Agriculture, Beijing, China

**Keywords:** Microbiome, Metagenomics

## Abstract

The dominant bacteria in the hindgut of calves play an important role in their growth and health, which could even lead to lifelong consequences. However, the identification of core probiotics in the hindgut and its mechanism regulating host growth remain unclear. Here, a total of 1045 fecal samples were analyzed by 16S rRNA gene sequencing from the 408 Holstein dairy calves at the age of 0, 14, 28, 42, 56, and 70 days to characterize the dynamic changes of core taxa. Moreover, the mechanisms of nutrient metabolism of calf growth regulated by core bacteria were investigated using multi-omics analyses. Finally, fecal microbiota transplantation (FMT) in mice were conducted to illustrate the potential beneficial effects of core bacteria. Four calf enterotypes were identified and enterotypes dominated by *Bifidobacterium* and *Oscillospiraceae_UCG-005* were representative. The frequency of enterotype conversion shifted from variable to stable. The close relationship observed between phenotype and enterotype, revealing a potential pro-growth effect of *Bifidobacterium*, might be implemented by promoting the use of carbohydrate, activating the synthesis of volatile fatty acids, amino acids and vitamin B6, and inhibiting methane production in the hindgut. The FMT results indicated the beneficial effect of *Bifidobacterium* on host growth and hindgut development. These results support the notion that the *Bifidobacterium*-dominated fecal microbiome would be an important driving force for promoting the host growth in the early life. Our findings provide new insights into the potential probiotic mining and application strategies to promote the growth of young animals or improve their growth retardation.

## Introduction

The importance of gastrointestinal tract (GIT) microorganisms to host health and metabolism is generally well recognized^1^. In ruminants, the effect of the microbiota might be amplified because the rumen houses highly abundant, diverse microbes that ferment plant fiber into volatile fatty acids (VFAs), providing 70–80% of their energetic requirements for maintenance and production^2^. Hence, to continually improve agricultural production efficiency, the ruminal microbial community and function of adult cows have been studied extensively^3–5^. Calves in the early stages of life do not have a fully developed rumen and the hindgut microbiome might play an important role in regulating the growth and development of calves. Although the food digestion pattern of calves is close to that of monogastric animals, which rely on a combination of host enzymes and symbiotic bacteria for digestion, they still have unique digestive characteristics compared with other animals, including specific bacteria, metabolites, and the direction of microbial evolution. Some studies on the temporal dynamics of the hindgut microbiota in calves have reported that dietary strategies^5^ and fecal microbiota transplantation (FMT)^6^ can significantly change the microbial community structure and thus improve calf growth performance and health. More importantly, the growth performance (body weight [BW] and average daily gain [ADG]) of calves in the preweaning and prepubertal periods is positively associated with their ability to produce milk in adulthood^7,8^, suggesting that the calf hindgut microbiota has a profound impact on long-term and even lifelong performance. Although these studies have expanded our knowledge, the representative characteristics of the calf hindgut microbiota in early life are lacking due to experimental specificity, small numbers of experimental animals, and possible individual variations. Our understanding of the differences in metabolic functions in the hindgut microbiota and core bacteria of calves with different phenotypes is also limited.

Enterotyping, which was first proposed to summarize the hindgut microbiota characteristics in humans^9^, is an effective method for stratifying populations, revealing a general overview of inter-individual differences in the gut microbial community. In subsequent studies, bacterial enterotype analysis was gradually extended to other mammals, such as chimpanzees^10^, swine^11^, and buffalo^12^. The results of all such studies have confirmed that the enterotype classification in adult hosts is dominated by critical bacterial taxa and relatively conservative regarding short-term disturbances^13,14^. However, compared with the redundant microbiota of adult dairy cows, the hindgut microbiota of calves in early life (from birth to weaning) is highly variable with age, and can be easily disturbed by diet^15^, resulting in unclear trends and timing of the transition between enterotypes in individual calves. Therefore, it is necessary to systematically study and understand the factors driving formation of hindgut enterotypes of pre-weaning calves, as well as the effects of the final enterotype on growth performance and physiological metabolism. Deeper understanding of these processes could provide important insights into the adaptability of young animals and their microecosystems to dietary changes.

Therefore, in this study, we aimed to answer the following questions. Which are the core bacteria that contribute to the growth of calves, and what are the related potential mechanisms? The 16S rRNA gene sequencing was used to identify the fecal core bacteria of calves across the ages. Multi-omics analyses (fecal metagenomics, fecal metabolomics and serum metabolomics) were conducted to reveal the mechanism of fecal core bacteria regulating the calf growth. The FMT in mice further emphasized the effect of core probiotics in calf feces on growth promotion.

## Results

### Calf sample characteristics

A large cohort of 408 Holstein dairy calves (150 female and 258 male) were enrolled in this study, from which a total of 1045 fecal samples were collected at birth (day 0), and at 7, 35, 56, and 70 days of age (Supplementary Fig. 1). Each sample was subjected to 16S rRNA sequencing to examine the microbial community and diversity. Fecal metagenomics, fecal metabolomics, and serum metabolomics were also performed for the samples from 20 calves selected randomly in the two different enterotype groups of 70 days of age (*n* = 10 per group). The integration of these omics could reveal the mechanism of microbial regulation of host development at the level of metabolic function.

### Landscape of age-related microbial changes in calves

**Fig. 1:**
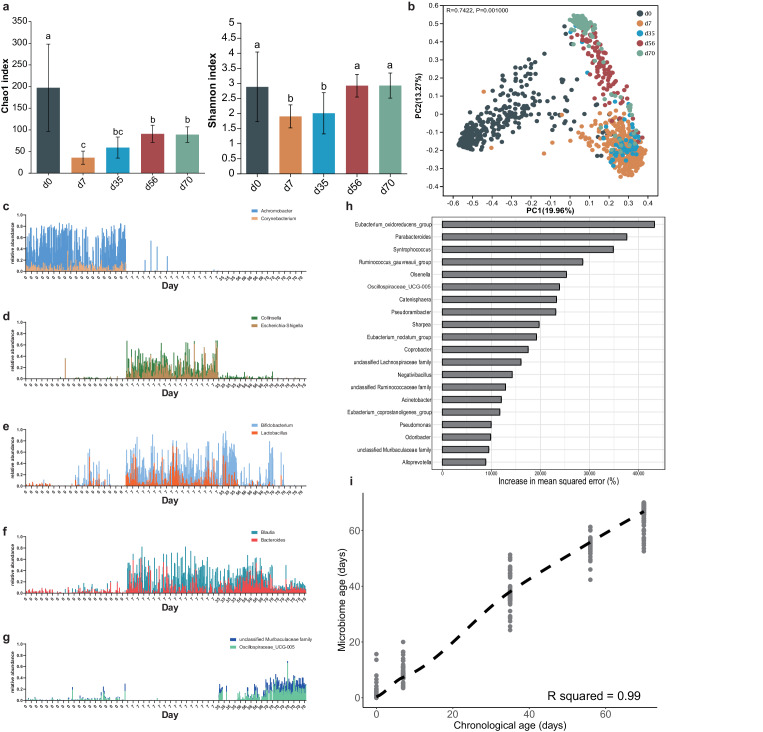
Dynamics of microbial diversity in the calf hindgut. **a** Alpha diversity of fecal microbiota at different time points. The significance was assessed using Kruskal–Wallis test. Different superscripts of English letters represented the significant differences between groups (*p* < 0.05). The error pounds in the bar charts were represented by mean ± SD. **b** Beta diversity of the gut microbiota of calves at different ages. Significance was assessed using ANOSIM. **c** Relative abundance of genera *Achromobacter* and *Corynebacterium*. **d** Relative abundance of genera *Collinsella* and *Escherichia-Shigella*. **e** Relative abundance of genera *Bifidobacterium* and *Lactobacillus*. **f** Relative abundance of genera *Blautia* and *Bacteroides*. **g** Relative abundance of genera *unclassified Muribaculaceae family* and *Oscillospiraceae_UCG-005*. The total samples were generally distributed according to age and the samples of the same age were arranged in the order of actual collection time on x-axis. **h** Top 20 age**-**related bacteria of the calf hindgut based on the mean square error of predictions (%IncMSE). **i** The distinct capability for age prediction based on the gut microbiota of calves from 0 to 70 days of age.

The information of 16S rRNA sequencing showed that ~50 million high-quality sequences were observed with 46422 ± 16249 (mean ± SD) reads per fecal sample. The results revealed the significant temporal characteristics in the calf microbiota. The highest values of microbial richness (Chao1) and diversity (Shannon) indexes were observed on the day of birth (Fig. 1a, Kruskal–Wallis test, *p* < 0.05). Interestingly, alpha diversity dropped significantly, reaching the lowest value at day 7, and increasing with age thereafter to day 56. No significant differences in alpha diversity were found between 56 and 70 days of age. Regarding beta diversity based on the principal coordinate analysis, distinct clustering of fecal microbiota by age group was observed (Fig. 1b and Supplementary Table 1, ANOSIM, *p* = 0.001). Specifically, the bacterial community at birth showed the biggest distance from those of the other age groups of calves. The bacterial communities of calves at 56 and 70 days of age were also significantly distinguishable from those at day 35.

At the genus level, we further identified the dominant bacteria at each time point. *Achromobacter* and *Corynebacterium* showed high abundances at birth, but had almost completely disappeared at the subsequent time points (Fig. 1c). *Collinsella* and *Escherichia-Shigella* were the specific dominant genera at 7 days of age (Fig. 1d). Notably, beneficial bacteria *Bifidobacterium* and *Lactobacillus* were the most abundant genera at 7 and 35 days of age, maintaining high abundances in some samples of 70-day-old calves (Fig. 1e). *Blautia* and *Bacteroides* became the dominant bacteria at 7 days, and retained their advantage to the age of 70 days (Fig. 1f). Genera *unclassified Muribaculaceae family* and *Oscillospiraceae_UCG-005* as dominant bacteria showed high abundances in subsets of samples from 35 and 56-day-old calves, becoming ubiquitous at 70 days of age (Fig. 1g). *Bacteroides*, *Olsenella*, *Faecalibacterium*, *Streptococcus*, and *Sharpea* were also found in high abundance in the calf hindgut (Supplementary Fig. 2).

By identifying age-associated bacteria, producer could make targeted interventions to promote faster maturation of calf hindgut microbiome. Here, the random forest machine learning algorithm was applied to regress the relative abundances of bacteria in the feces against the subjects’ chronological ages. We evaluated regression performance using mean absolute error. The predictive result was supported by the consistency of observed high-abundance signatures of microbes with high feature importance scores, such as S*yntrophococcus*, *Olsenella*, *Oscillospiraceae_UCG-005*, and *Sharpea* (Fig. 1h). The model accuracy (*R*^2^) for age predicted by fecal microbiota reached 0.99 (Fig. 1i).

### Classification of the early calf hindgut microbiota based on enterotyping

**Fig. 2:**
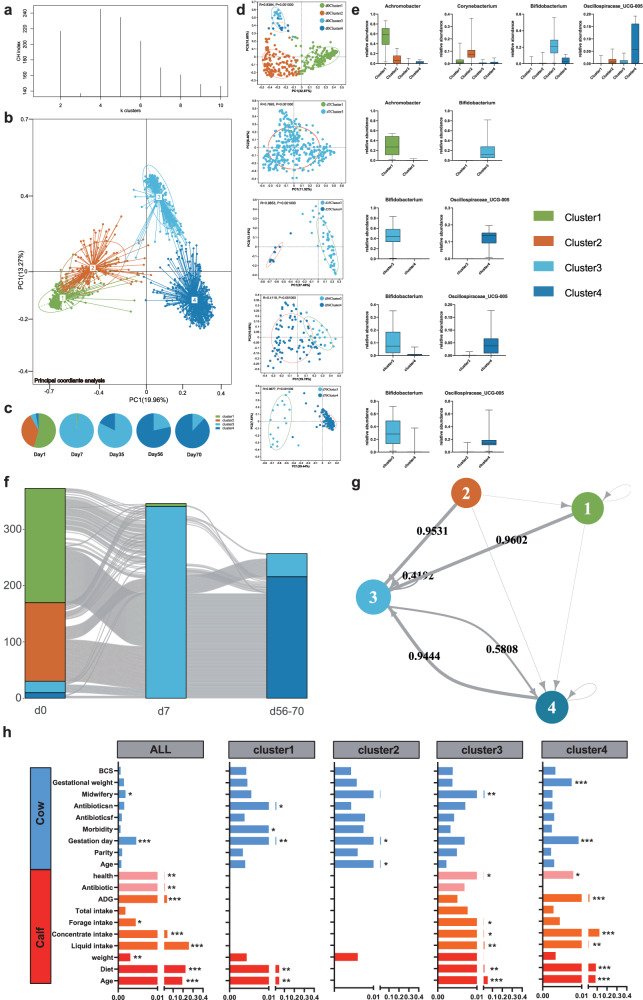
Characteristics of four calf enterotypes associated with age and diet. **a** CH index of enterotype robustness. **b** Principal coordinate analysis of the four differential clusters based on all samples. **c** Proportions of the four clusters at different calf ages. **d** Distribution of enterotypes at different calf ages. The significance between groups was assessed using ANOSIM. **e** Box plots of relative abundance of the major bacterial contributor of each enterotype. The five horizontal lines in the box plots represented the upper limit, the upper quartile, the median, the lower quartile, and the lower limit. **f** Sanky diagram of transitioning of the four enterotypes at different calf ages. **g** Markov chain analysis with subject-independent transition probabilities among the four enterotypes. **h** PERMANOVA analysis of the amount of variance explained by multiple factors influencing the gut microbiota. Only the significant differences were marked (*p < 0.05, **0.01< *p* < 0.05, ***p < 0.001).

To further classified the subtypes of calf microbiota and determine their related core bacteria, enterotype analysis was conducted. As the result, the hindgut microbiota has been classified into several stable enterotypes, defined by the abundance of key taxa. However, the paradigm of the bacterial community type has remained unclear in calves. According to the Calinski Harabasz (CH) index of partitioning around medoids (PAM), the microbial profiling demonstrated an optimal number of four clusters (*k* = 4) showing the best robustness in the calf fecal community (Fig. 2a, b). To better understand the four enterotypes and to determine the temporal distributions of bacteria in the calf microbiota in the first 70 days of life, we explored the emergence windows of each cluster (Fig. 2c). Clusters 1 (*n* = 209) and 2 (*n* = 140) were the two main enterotypes of calves at birth and were represented by relatively high abundances of genera *Achromobacter* and *Corynebacterium*, respectively. However, these two enterotypes of fecal samples disappeared rapidly after birth. Cluster 3 (*n* = 458) became the most predominant enterotype at 7 days of age and were dominated by the genus *Bifidobacterium* in high abundance. For the period from 35 to 70 days of age, clusters 3 and 4 (*n* = 238) were the representative enterotypes; with age, the proportion of cluster 3 decreased and that of cluster 4 increased. *Oscillospiraceae_UCG-005* became the dominant genus of bacteria in the microbial community of cluster 4 (Fig. 2d, e and Supplementary Fig. 3).

We also investigated the transition process of hindgut enterotypes across different ages in the early stage of calves. As shown in Fig. 2f, a general inter-enterotype variation tendency was observed throughout the entire period of early life. Notably, the most frequent transition occurred in the first week of early life, during which nearly all of the calves in clusters 1 and 2 shifted to cluster 3. From day 7 to post weaning (days 56–70), about 85% of calves in cluster 3 transitioned to cluster 4 and the remainder showed no variation between clusters 3 and 4, suggesting a decelerating trend of enterotype transition. Next, we performed Markov chain analysis, a method used to establish the model of enterotype transition probabilities, to quantify the relationships among different microbiota enterotypes (Fig. 2g). Specifically, clusters 1 and 2 showed high frequencies of transitioning to cluster 3, with probabilities of 0.95 and 0.96, respectively. Furthermore, cluster 3 was quite conserved, with a self-transition probability of 0.42. There was close transition interaction between clusters 3 and 4, with probabilities reaching 0.58 for the transition from cluster 3 to 4, and 0.94 for cluster 4 to 3.

To identify the possible factors influencing hindgut enterotype transition, we analyzed both calf and maternal factors using permutational multivariate analysis of variance (PERMANOVA) (Fig. 2h). In general, age and diet were the top 2 factors shaping the hindgut microbiota in all four enterotypes. The intake of different dietary types (liquid, concentrate, and forage) also significantly impacted the hindgut microbiota. Intriguingly, the enterotype transition may have occurred in response to certain maternal factors including gestational body condition score (BCS), gestational weight, midwifery (the delivery process that requires the midwife to help correct the fetal position or give external force), whole-life antibiotic treatment (antibioticcsf), gestational antibiotic treatment (antibioticcsn), gestational morbidity (morbidity), gestational day, parity and age. Specifically, the gestational day had significant effects on clusters 1, 2, and 4, whereas morbidity and antibiotic treatment in the gestation period of dams influenced the hindgut microbiota of cluster 1. Gestational weight had a profound effect on the microbial community of cluster 4.

The co-occurrence networks of bacteria were established (Supplementary Fig. 4) to better understand the interactions among hindgut bacteria in the different enterotypes. For the network encompassing the entire evaluation period (Supplementary Fig. 4a), we identified signatures of each enterotype clustered by close associations using linear discriminant analysis (LDA) effect size (LEfSe), with cutoffs of LDA > 2 and *p* < 0.05 (Supplementary Fig. 5). In the cluster 1 network (Fig. S6B), most correlations between bacteria were negative, and the signature taxa were loosely distributed throughout the network. However, with the enterotype transition from cluster 2 to clusters 3 and 4 (Supplementary Fig. 4b–e), there were more positive interactions among the bacteria in each network, and the signatures of each enterotype had higher degrees of aggregation (Supplementary Fig. 4f). The network parameters proved that the network complexity of clusters 3 and 4 was higher than that of clusters 1 and 2, with lower node numbers and higher edge numbers. Moreover, anti-interference testing revealed that cluster 4 exhibited the highest network stability, followed by clusters 3, 2, and 1 (Supplementary Fig. 4g).

We further analyzed functional variations of the four enterotypes using PICRUSt functional prediction to investigate the potential effects of enterotype evolution and transition on calf growth and development. Consistent with the microbial communities of the enterotypes, functional profiling also showed four distinct clusters (Supplementary Fig. 6b, ANOSIM, *p* = 0.0001). The clusters 3 and 4 exhibited a relative similar functional structure due to a large

number of overlapping samples. Conversely, clusters 1 and 2 showed a considerably distinct difference in functional distance, despite both being dominant clusters at the birth of calves.

Next, we focused on the metabolic pathways underlying the remarkable specificity of enterotype with age. At level 2 of the Kyoto Encyclopedia of Genes and Genomes (KEGG) database, a prevalent difference of 46 functional categories was observed between the enterotypes (Supplementary Fig. 6a, Kruskal–Wallis test, *p* < 0.05). We further compared pathways associated with the synthesis and metabolism of substances at level 3. As shown in Supplementary Fig. 4h, “butanoate metabolism” and “glyoxylate and dicarboxylate metabolism” were enriched in clusters 1 and 2 and depleted in clusters 3 and 4. In contrast, pathways associated with amino acid metabolism and carbohydrate metabolism showed higher abundance in clusters 3 and 4 than in clusters 1 and 2. Among these, the pathways of “starch and sucrose metabolism” and “amino sugar and nucleotide sugar metabolism” in cluster 3 were the most abundant. Ultimately, considering the advantages of gut microbiota in carbohydrate decomposition and amino acid and fatty acid metabolism, we selected three related representative pathways in which to explore temporal changes across all the calves (Supplementary Fig. 4i). As expected, the pathways of “starch and sucrose metabolism” and “alanine, aspartate and glutamate metabolism” increased in abundance with age, whereas “butanoate metabolism” showed the opposite pattern. We further grouped calves by enterotype to track the functional changes of each enterotype. The abundance of the “starch and sucrose metabolism pathway” increased with age in clusters 1 and 3, but decreased with age in cluster 4. The abundance of the “butanoate metabolism” pathway significantly decreased with age in clusters 1 and 3, but showed no changes in cluster 4. These three clusters all exhibited a consistent upward trend in abundance of the pathway of “alanine, aspartate, and glutamate metabolism”.

### An enterotype dominated by Bifidobacterium shaped favorable phenotypes of calves

**Fig. 3:**
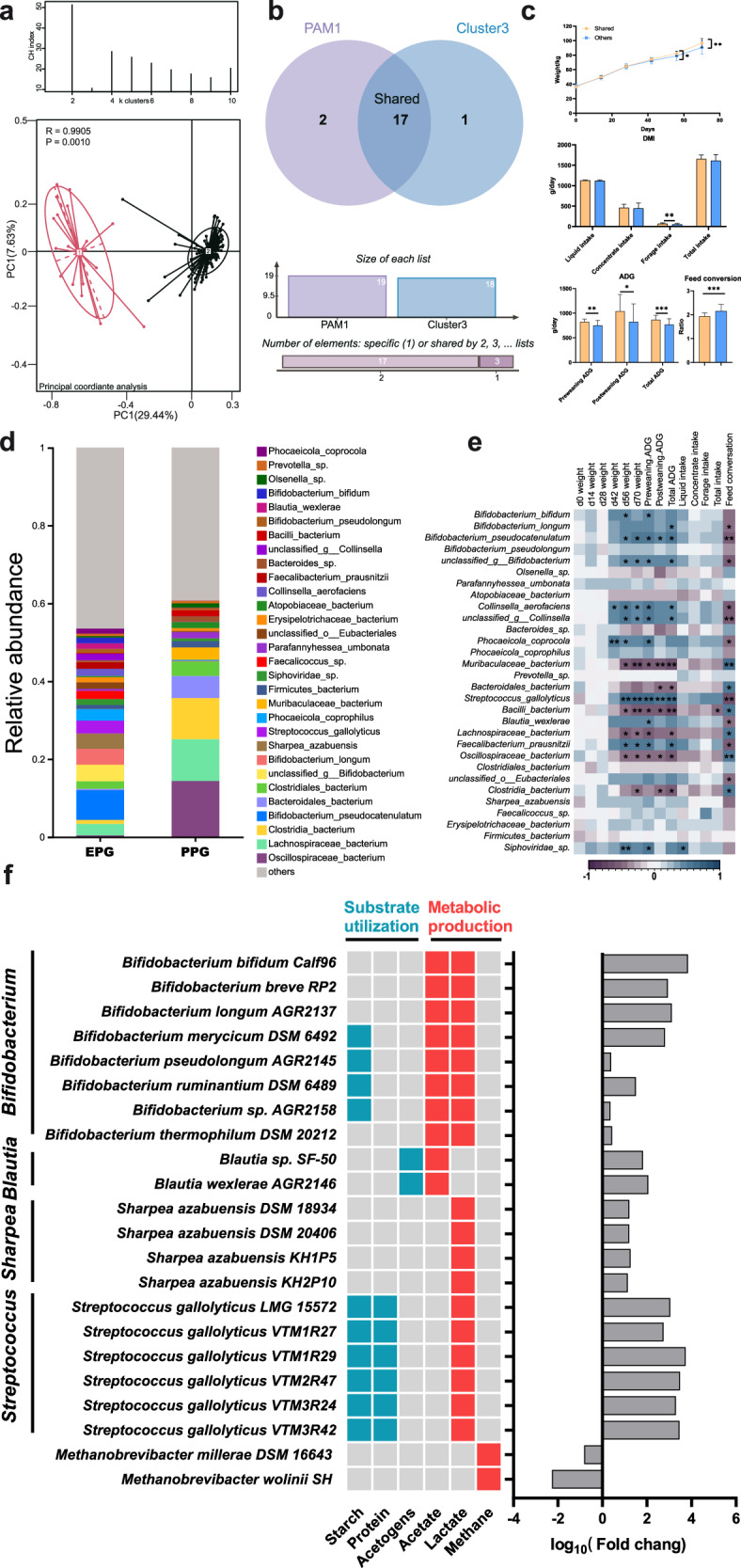
Contribution of the *Bifidobacterium*-dominant enterotype to favorable calf phenotypes. **a** CH index of enterotype robustness and Principal coordinate analysis of the two differential clusters based on samples taken at 70 days of age. The significance was assessed using ANOSIM. **b** Wayne diagram showing the intersection of cluster 3 and PAM 1. **c** The comparison of growth performance in calves between the Shared and Others groups. The significance was assessed using one-way ANOVA. The error pounds in the charts were represented by mean ± SD. **d** Composition of the gut microbiota at the species level in EPG and PPG. **e** Spearman correlation analysis between species and phenotypes. **f** Identification of cultured strains in the Hungate1000 collection. Feed conversion = Average daily intake/average daily gain. **c**, **e** Only the significant differences were marked (*p < 0.05, **0.01< *p* < 0.05, ***p < 0.001).

We also explored the relationships between different enterotypes and their corresponding phenotypes, focusing on the 70-day-old calves due to their detailed growth data and relative bodily maturity. Although the 70-day-old calves were classified into two enterotypes (clusters 3 and 4) in the previous section, to avoid the influence of other age samples on the enterotyping, we performed a separate enterotype analysis of fecal samples from 70-day-old calves. In agreement with the above results, two enterotypes (PAM 1 and PAM 2) exhibited the most accuracy with the highest CH indexes (Fig. 3a). As shown in the Wayne diagram, most of the samples in cluster 3 (17/18) and PAM 1 (17/19) were shared, suggesting the high stability of enterotype classification in the 70-day-old calves (Fig. 3b). We then compared the phenotypes among the shared calves (cluster 3/PAM 1) and all other 70-day-old calves, which revealed that the shared calves had better growth performance, including higher body weight, ADG, and feed conversion (Fig. 3c, one-way ANOVA, *p* < 0.05). Regarding serum indicators, the concentration of albumin (ALB) was higher and that of glutathione peroxidase (GSH-PX) was lower in the shared calves, with no other indicators showing differences between these two groups (Supplementary Fig. 7). To further clarify the mechanism of enterotype-mediated regulation of host growth performance, 10 calves were selected randomly from each group (shared and others), and fecal samples and blood samples were collected for multi-omics sequencing (fecal metagenomics, and fecal and serum metabolomics). We defined the subgroup of 10 shared calves as the excellent phenotype group (EPG) and the subgroup of 10 other calves as the prevalent phenotype group (PPG). These 20 fecal samples were sequenced ~2.1 billion quality-filtered reads were obtained after removing host contamination and ~6.6 million contigs were assembled. A distinct distance of hindgut microbiome structure was observed between EPG and PPG (Supplementary Fig. 8a, ANOSIM, *p* = 0.001), with a significantly higher alpha diversity in PPG as measured by the Chao1 index (Supplementary Fig. 8b, Wilcoxon rank-sum test, *p* = 0.0001). Next, we examined the microbial composition of each group at the species level (Fig. 3d). In EPG, the dominant species were *B. pseudocatenulatum*, *B. longum*, *B. bifidum*, and *B. pseudolongum* belonging to genus *Bifidobacterium*, *S. gallolyticus* belonging to genus *Streptococcus*, and *S. azabuensis* belonging to *Sharpea*. In PPG, the dominant species were *Oscillospiraceae bacterium*, *Lachnospiraceae bacterium*, *Clostridia bacterium*, and *Bacteroidales bacterium*. Correlation analysis indicated that *S. gallolyticus*, *C. aerofaciens*, *B. pseudocatenulatum*, and *F. prausnitzii* were significantly positively associated with phenotypic features including body weight, ADG, and feed conversation (Feed conversion = Average daily intake/average daily gain). Conversely, the *O. bacterium*, *L. bacterium*, *Bacilli bacterium*, and *Muribaculaceae bacterium* were significantly negatively associated with these features (Fig. 3e, Spearman analysis, *p* < 0.05). Hungate1000 collection^16^, a database of bacterial and archaeal species isolated and cultured from the GIT of a variety of ruminants, was used to further deepen our microbial taxa identification at the strain level (Fig. 3f). A total of 406 strains were identified, 193 of which showed significant differences between the two groups. We focused on strains belonging to those species known to be the main hosts of critical metabolic functions. *Bifidobacterium* members, including *B. bifidum Calf96*, *B. RP2*, and *B. longum AGR2137*, *Blautia* members including *B. sp. SF-50* and *B. wexlerae AGR2146*, *Sharpea* members including *S. azabuensis DSM 18934* and *S. azabuensis DSM 20406*, and *Streptococcus* members including *S. gallolyticus LMG 15572* and *S. gallolyticus* VTM1R29, were the dominant taxa in EPG. The *Bifidobacterium* members favored starch utilization and production of acetate and lactate. *Streptococcus* members showed the ability to use starch and protein and produce lactate. Two methanogenic strains of the genus *Methanobrevibacter*—*M. millerae DSM 16643* and *M. wolinii SH*—were identified as representative taxa in PPG.

### Characteristics of metabolic functions of the hindgut microbiome in phenotypically favorable EPG calves

**Fig. 4:**
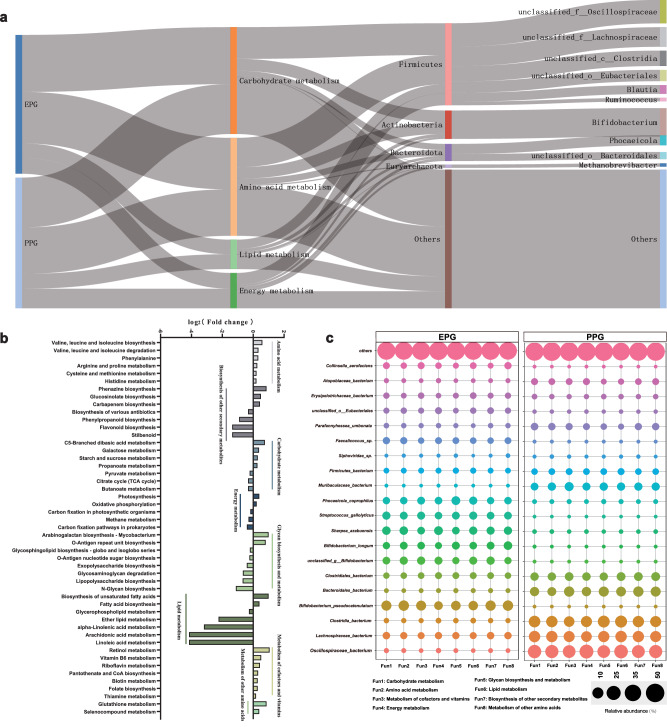
Differences in KEGG metabolic functions encoded by the gut microbiomes of EPG and PPG. **a** Sankey diagram connecting the four major metabolic pathways (2nd column) from EPG and PPG (1st column) to the predicted bacterial hosts at the phylum (3rd column) and genus (4th column) levels. **b** Significant between-group differences in KEGG pathways related to metabolism of the fecal microbiota. The significance was assessed using Wilcoxon rank-sum test. **c** Bubble plots depicting differences in metabolic functions of the microbial hosts at the species level.

Metagenomic functional analysis was performed to characterize the functions performed by hindgut core microbes of calves. The pathways involved in the organic substance metabolic process showed significant differences between EPG and PPG (Fig. 4b, Wilcoxon rank-sum test, *p* < 0.05). Specifically, EPG calves showed higher abundances of amino acid metabolism pathways, including “valine, leucine, and isoleucine biosynthesis”, “phenylalanine, tyrosine, and tryptophan biosynthesis”, “histidine metabolism”, “arginine and proline metabolism”, and “glutathione metabolism”. The pathways related to vitamin metabolism, such as “retinol metabolism”, “vitamin B6 metabolism”, “riboflavin metabolism”, and “biotin metabolism”, showed similar trends in EPG. Additionally, the carbohydrate metabolism pathways of “starch and sucrose metabolism”, “galactose metabolism”, and “propanoate metabolism”, and the lipid metabolism pathways of “biosynthesis of unsaturated fatty acids” and “fatty acid biosynthesis” were abundant in EPG. In PPG, the carbohydrate metabolism pathways of “pyruvate metabolism“, “butanoate metabolism”, and “citrate cycle”, and the energy metabolism pathways of “methane metabolism”, “arabinogalactan biosynthesis”, and others were higher in abundance. Next, we tracked the microbial hosts of the four major metabolic functions (amino acid, carbohydrate, energy, and lipid metabolism) through metagenomic assembly (Fig. 4a). Firmicutes, Actinobacteria, and Bacteroidota were the dominant phyla harboring the functional genes involved in the major metabolic activities. The *Methanobrevibacter* belonging to phylum Euryarchaeota was found to be an important host of energy metabolism. At the species level, distinct between-group differences in the metabolic functions of microbial hosts were observed. Consistent with the microbial community composition, *Bifidobacterium* members were the primary hosts in EPG, including *B. pseudocatenulatum*, *B. longum*, and unclassified *Bifidobacterium*. Additionally, *S. azabuensis*, *S. gallolyticus*, and *Phocaeicola coprophilus* were also dominant host bacteria in EPG. In PPG, *O. bacterium*, *L. bacterium*, *C. bacterium*, and *B. bacterium* were the main microbial hosts (Fig. 4c). We also analyzed the microbial host composition of the top 10 metabolic sub-pathways and found that it was similar to that of the above metabolic functions (Supplementary Fig. 9). In the “methane metabolism” sub-pathway, *Methanobrevibacter_sp*. was dominant, explaining its important contribution to energy metabolism.

A total of 3005 genes with significant differences were identified between the two groups: 1078 genes enriched in EPG and 1927 genes enriched in PPG. The KEGG enrichment analyses of these genes in each group were visualized using the KEGG Mapper. As shown in Supplementary Fig. 10a, the differential genes in EPG were enriched in lipid metabolism. Genes common to both groups were enriched in amino acid metabolism and carbohydrate metabolism. Specifically, the pathways of “biosynthesis of amino acid” and “fatty acid metabolism” were activated in EPG through the upregulation of genes related to biosynthesis of valine, leucine, histidine, and isoleucine, and elongation of fatty acid chains (Supplementary Fig. 10b, c). Conversely, methane metabolism-related genes were higher in abundance in PPG (Supplementary Fig. 10d).

**Fig. 5:**
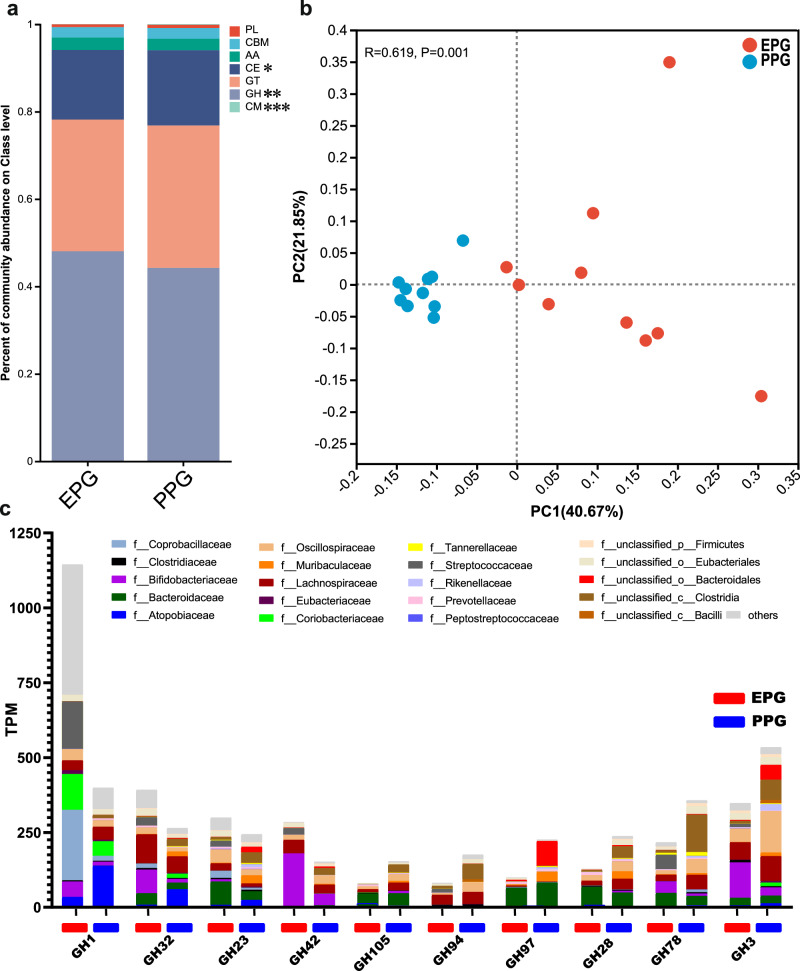
Distinct enrichment of CAZyme coding genes in the fecal microbiome of EPG calves. **a** Relative abundance of all CAZyme genes in EPG and PPG. Only the significant differences were marked (*p < 0.05, **0.01< *p* < 0.05, ***p < 0.001). **b** Principal coordinate analysis of the GH enzyme family in EPG and PPG. The significance was assessed using ANOSIM. **c** Abundance of the top 10 significantly different GH enzymes, with the predicted enrichment of the corresponding microbial hosts. TPM Transcripts Per Million. **a**, **c** The significance was assessed using Wilcoxon rank-sum test.

Next, considering that the decomposition of carbohydrates was an important characteristic of nutritional metabolism of microbiome, we focused on carbohydrate-active enzyme (CAZyme) in these two groups. Glycoside hydrolases (GHs) were the most abundant of the seven CAZyme classes, showing a distinct difference between EPG and PPG (Fig. 5a, ANOSIM, *p* = 0.009). Therefore, we further compared the abundances of GH members in the two groups, illustrating the top 10 GH members with significant differences in Fig. 5b (Wilcoxon rank-sum test, *p* < 0.05). Specifically, EPG exhibited higher abundances of GH1, GH32, GH23, and GH42, which were mainly assigned to families *Bifidobacteriaceae*, *Coprobacillaceae*, *Lachnospiraceae*, and *Streptococcaceae*. In PPG, the abundances of GH3, GH78, GH28, GH97, GH94, and GH105 were higher and were assigned to families *Bacteroidaceae*, *Oscillospiraceae*, *Lachnospiraceae*, and *unclassified_Clostridia* (Fig. 5c).

### Significant metabolic profile differences between EPG and PPG calves

**Fig. 6:**
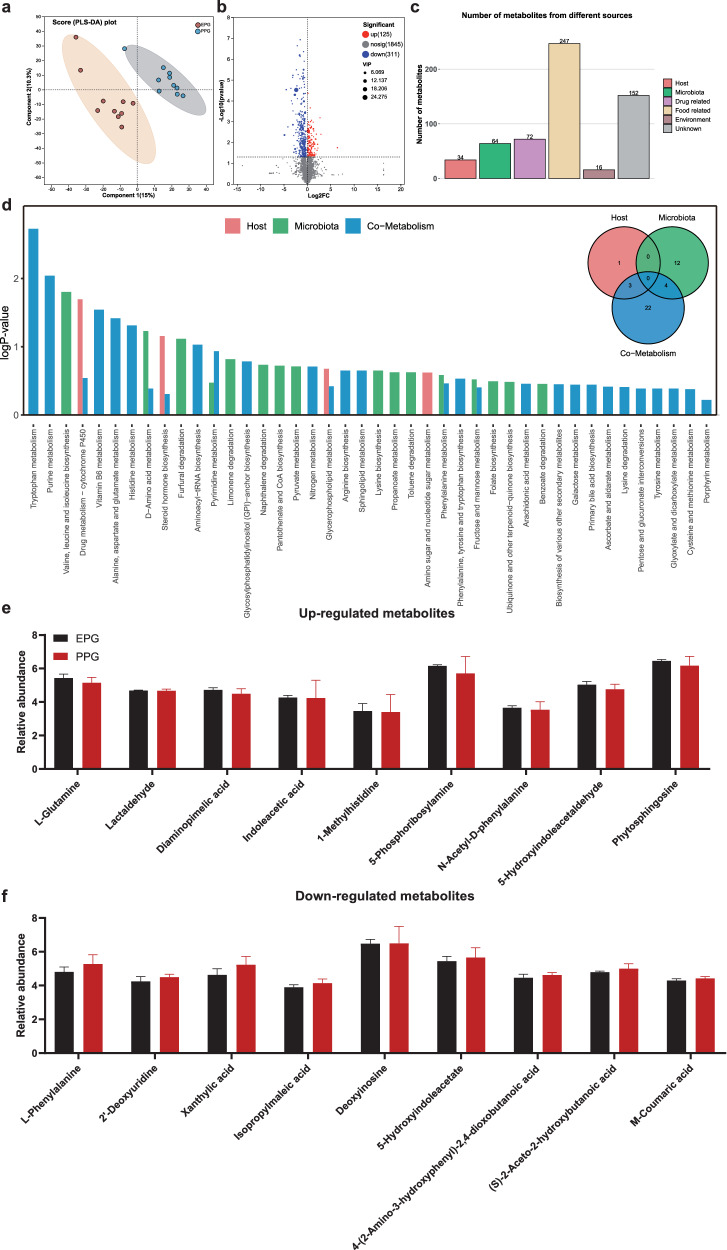
Fecal metabolic profiles of EPG and PPG. **a** PLS-DA of the fecal metabolomes between EPG and PPG calves. **b** Volcano map of metabolites identified by fecal metabolomics. **c** Identification of fecal metabolites from different sources. **d** Metabolic pathway enrichment analysis of different categories of metabolites from different sources. Abundance of upregulated (**e**) and downregulated (**f**) metabolites involved in the enrichment of metabolic pathways. The error pounds in the bar charts were represented by mean ± SD.

Metabolomic analyses were also performed on feces and blood samples from EPG and PPG calves, which allowed us to determine the differences between the two groups from the perspective of host and microbial metabolic profiles. Partial least squares-discriminant analysis (PLS-DA) showed distinct metabolic profiles of feces from EPG and PPG (Fig. 6a). A total of 2281 fecal metabolites were quantified, of which 402 differential metabolites were identified: 118 upregulated metabolites and 284 downregulated metabolites (Fig. 6b). Given the close interaction between the hindgut microbiota and the host, we conducted MetOrigin analysis^17^ to better characterize the origin of these metabolites (host, bacteria, or both). The results showed that 27 metabolites were derived from the host, 59 metabolites were from the microbiota, and 22 metabolites were shared by the two; the remaining 381 metabolites were from other sources (drugs, food, environment, and unknown) (Fig. 6c). Functional enrichment analysis of the metabolites from host, microbiota, and both sources (Fig. 6d) revealed a total of 48 metabolic pathways comprising 30 shared pathways, 21 microbiota-specific pathways, and four host-specific pathways. Consistent with the results of microbial functional enrichment, the pathways of “valine, leucine, and isoleucine biosynthesis”, “vitamin B6 metabolism”, “sphingolipid metabolism”, “D-amino acid metabolism”, and “alanine, aspartate, and glutamate metabolism” were enriched by multiple differential metabolites. These include upregulated L-glutamine, lactaldehyde, indoleacetic acid, and 5-phosphoribosylamine, (Fig. 6e) and downregulated L-phenylalanine, isopropylmaleic acid, 5-hydroxyindoleacetate, and (S)-2-aceto-2-hydroxybutanoic acid (Fig. 6f). The blood metabolomics also revealed remarkable between-group differences in metabolic compounds (Supplementary Fig. 11a). In total, 302 significantly differential blood metabolites were identified (114 upregulated and 188 downregulated) (Supplementary Fig. 11b), among which 33 and 57 metabolites were derived from the microbiota and the host, respectively (Supplementary Fig. 11c). The pathways of “lysine biosynthesis”, “valine, leucine and isoleucine degradation”, “arachidonic acid metabolism”, “phenylalanine, tyrosine and tryptophan biosynthesis”, and “tryptophan metabolism” (Supplementary Fig. 11d) were enriched by differential metabolites, including PE(22:6(4Z,7Z,10Z,13Z,16Z,19Z)/16:1(9Z)), PC(15:0/22:4(7Z,10Z,13Z,16Z)), allysine, succinic acid, and methylmalonic acid (Supplementary Fig. 11e, f).

### Multi-omics analysis of the active metabolic pathways in the EPG calf hindgut

**Fig. 7:**
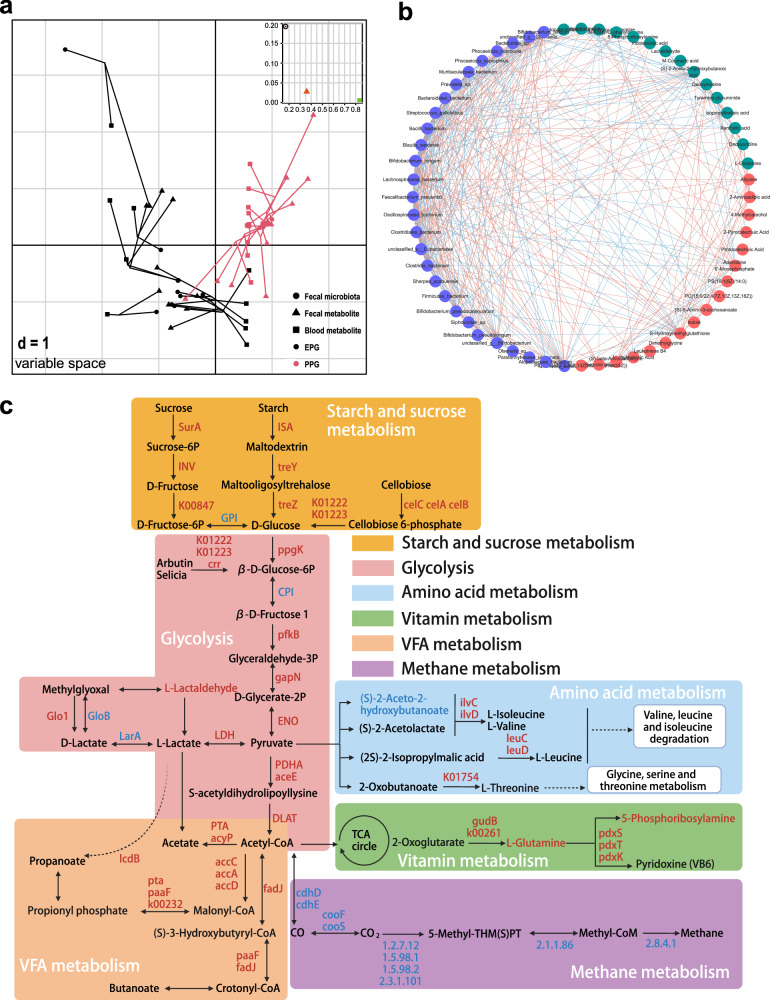
Joint analysis of multi-omics data. **a** MCIA of the fecal microbiome, fecal metabolites, and blood metabolites in EPG and PPG. **b** Network analysis of interactions among the fecal microbiome, fecal metabolites, and blood metabolites. **c** Integration of different metabolic pathways involved in starch and sucrose metabolism, glycolysis, amino acid metabolism, vitamin metabolism, VFA metabolism, and methane metabolism.

Joint analysis of multi-omics data was also performed to understand how the fecal microbiome affected the growth performance of the host through microbial metabolism and co-metabolism with the host. Using multiple co-inertia analysis (MCIA)^18^ projection plots, we observed close co-relationships and trends between three high-dimensional datasets and a distinct division between EPG and PPG. We then calculated the pseudo-eigenvalues associated with the first two principal components (PCs) of each dataset, which could explain the contribution of each dataset to the total variance and correlation between the datasets. Fecal microbiota and blood metabolites had the highest degree of explanation values on Axis 1 and Axis 2, respectively, with fecal metabolites showing a stronger correlation with the fecal microbiota than with blood metabolites (Fig. 7a). The co-occurrence network also indicated potential associations between the dominant microbes and critical metabolites (Fig. 7b). Combined with the enrichment pathways shared by the microbiome and metabolomics data, we identified and described the predominant metabolic functions regulated by the hindgut microbiome of calves. As shown in Fig. 7c, genes involved in the degradation of exogenous carbohydrates (e.g., *SurA*, *INV*, *K00847*, *treY*, *treZ*, *celC*, and *celB*), including sucrose, starch, and cellobiose, were upregulated in EPG. In the subsequent glycolysis process, most of the related genes (*ppgk*, *pfkB*, *gapN*, *aceE*, and others) and one metabolite L-lactaldehyde were increased, whereas only a few related genes (*CPI*, *GloB*, and *LarA*) were decreased in abundance in EPG. Similarly, VFA and amino acid metabolism were activated in EPG, including the upregulated genes *ilvC*, *ilvD*, *leuC*, *leuD*, *accC*, *paaF*, and *IcdB*. Notably, genes (*gudB*, *k00261*, *pdxS*, *pdxT*, and *pdxK*) and metabolites (L-glutamine and 5-phosphoribosylamine) related to synthesis of vitamin B6 were also increased in EPG. Conversely, genes involved in methane metabolism (*cdhD*, *cdhE*, *CooF*, *cooS*, *EC1.2.7.12*, *EC 2.1.1.86*, *EC 2.8.4.1*, and others) were all upregulated in PPG.

### Transplantation of the Bifidobacterium-dominant EPG gut microbiota promoted the growth of mice

**Fig. 8:**
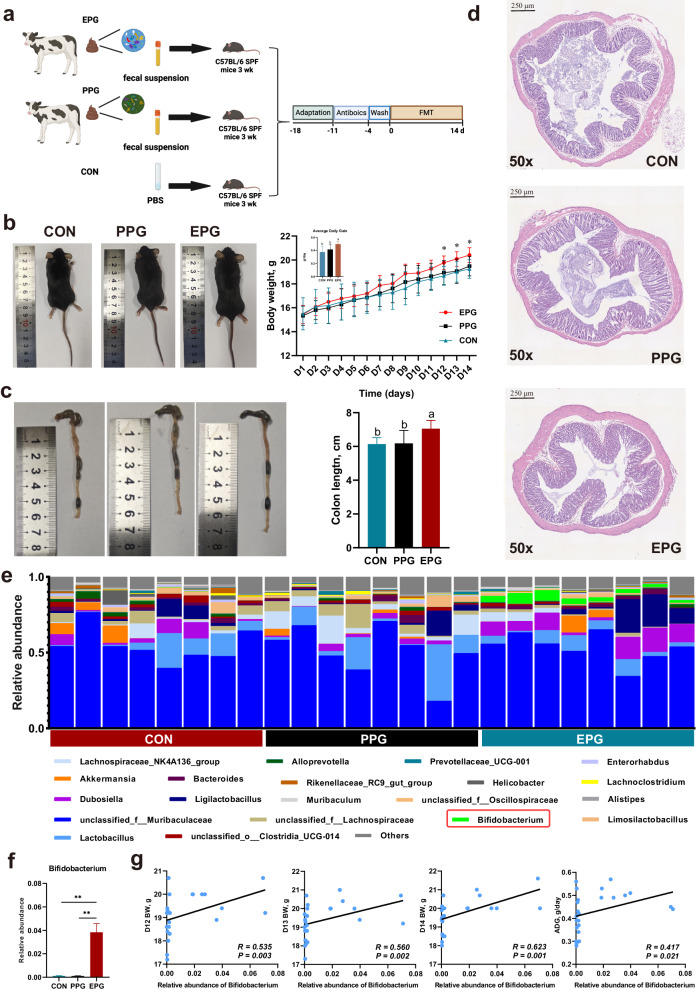
Effects of FMT from EPG and PPG calves on growth performance and colonic development of mice. **a** Experimental design diagram. **b** Body condition images (left) and growth performance data (right) of mice after FMT from each calf group. **c** Representative images (left) and length data (right) of the colon in each group. **d** Representative H&E staining (×50 magnification) of representative colonic sections from each group. Scale bar was 250 μm. **e** The microbial composition of fecal samples in each group of mice at the genus level. **f** The relative abundance of *Bifidobacterium* in each group. The significance was assessed using Kruskal–Wallis test. The error pounds in the bar charts were represented by mean ± SEM. **g** Linear regression relationship between *Bifidobacterium* abundance and growth performance of mice. **b**, **c** The significance was assessed using one-way ANOVA. Different superscripts of English letters represented the significant differences between groups (*p* < 0.05). The error pounds in the charts were represented by mean ± SD. **b**, **f** Only the significant differences were marked (*p < 0.05, **0.01< *p* < 0.05, ***p < 0.001).

According to the above analysis, we confirmed the close relationship between *Bifidobacterium* and calf phenotype, and evaluated the potential regulatory mechanisms. However, it was necessary to provide more direct evidences that *Bifidobacterium*-dominated fecal microbiome could promote the growth and development of the host (not only to calves), which was a prospective step for the development of universal probiotic preparations. Therefore, mice as the conventional animal model were used to conduct the FMT experiment (Fig. 8a). We observed that BW in the last 3 days of FMT was significantly higher in EPG mice than in PPG and CON mice. The EPG mice also showed a higher ADG (Fig. 8b, one-way ANOVA, *p* < 0.05). Anatomical comparison revealed that EPG mice had a larger body size and a longer colon length compared with the other groups (Fig. 8c, one-way ANOVA, *p* < 0.05). Furthermore, histological analysis revealed that EPG mice had a more complete and compact colonic epithelial structure and a shallower recess, suggesting a higher degree of colonic development (Fig. 8d). The phenotypic characteristics of these mice proved that the hindgut microbiota of EPG calves could promote the growth and gut development of another species of young animals. In addition, we also conducted 16S sequencing to detect the microbial composition in the feces of mice in each group. As shown in the Fig. 8e, f, *Bifidobacterium* was prevalent with higher abundance in the EPG mice compared with other groups (Kruskal–Wallis test, *p* < 0.05) and showed the significant correlations with ADG and the BW of the last 3 days of the experiment (Fig. 8g, Spearman analysis, *p* < 0.05), demonstrating the successful colonization of *Bifidobacterium* in the gut of EPG mice and its close correlation with growth performance.

## Discussion

The early colonization and evolution of the hindgut microbiota have profound effects on the growth and productivity of calves. Prior to this study, the hindgut core microbes in the early life of calves had not been fully summarized and the characteristics had not been deeply probed. This is the first study to integrate large-sample multi-omics analyses to identify the enterotypes of young ruminants, revealing how the dominant core bacteria of each enterotype regulated the hindgut metabolism to influence the growth phenotype. Our classification produced precise, dynamic calf enterotypes, highlighting that the *Bifidobacterium*-dominated microbiome improved calf growth by regulating the metabolism of substances in the hindgut.

Consistent with previous studies^19,20^, the meconium was distinguished from the feces of other growth stages by its high microbial diversity and unique composition. Diarrhea caused by pathogenic *Escherichia coli* infection is an important threat to the health of calves, especially newborn calves under 3 weeks of age^6,20,21^. Similarly, we also detected the high abundance of *Escherichia-Shigella* in the feces of our calves at the first week of age, suggesting a high susceptibility to diarrhea in calves at this stage. Fortunately, consistent with previous studies^22,23^, beneficial bacteria that persisted from 7 days of age to the post weaning phase were also observed, including *Bifidobacterium*, *Lactobacillus*, *Blautia*, and *Bacteroides*, suggesting that the microbial community of the calf hindgut gradually matures and becomes more diverse. Furthermore, we observed that *unclassified Muribaculaceae family* and *Oscillospiraceae_UCG-005*, which are reportedly enriched in animals with high-fiber diets and positively correlated with fatty acid concentrations^24,25^, were present at 35 days of age, becoming the dominant genera at 70 days of age. In this study, calves consumed more forage with increasing age, which might have provided an abundance of nutritional substrate for the proliferation of these two kinds of bacteria. Notably, even among individual calves of the same age, the compositions of the microbiota were not completely consistent, implying the existence of different gut types among calves. Additionally, studies in other livestock have confirmed that the various gut microbes could be used to predict host chronological age^26,27^. Indeed, in this study, we used a random forest regression model to identify a set of fecal microbes that predict calf age with high accuracy. Hence, it was unsurprising that some bacteria involved in nutrient digestion were correlated with calf age and physiological alteration.

In this study, although a total of four enterotypes were detected, only two (cluster 3 dominated by *Bifidobacterium* and cluster 4 dominated by *Oscillospiraceae_UCG-005*) were resident in the calf hindgut from birth to post weaning, and thus identified as representative enterotypes. The most variation in enterotypes occurred in the first week of life. During this period, clusters 1 and 2 (dominated by *Achromobacter* and *Corynebacterium*, respectively) rapidly transitioned to cluster 3. Although the proportion of cluster 3 bacteria continued to decrease and be replaced by cluster 4 bacteria with age, *Bifidobacterium* undeniably remained as the dominant bacteria in some of our post-weaning calves. In other studies, *Bifidobacterium* was also identified as one of the earliest colonizers in the gut of newborn calves^28^. Although its abundance in some individuals decreased with the ages, we still observed the prevalence of *Bifidobacterium* in the fecal microbiota of lactating dairy cow, and its abundance was positively correlated with cows’ healthy status^29^. These evidences indicated that *Bifidobacterium* was not unique to a specific age of host, but its abundance and prevalence varied among hosts of different ages, which might be attributable to host condition and dietary regime. As expected, the functional profiles of the enterotypes showed remarkable temporal characteristics. Even at the same time point, the pattern of metabolic function between clusters 3 and 4 was different, which might be involved in promoting phenotypic differentiation of calves.

Therefore, in calves at 70 days of age, we identified a significant association between enterotype and phenotype. Calves with the *Bifidobacterium* enterotype showed the best growth performance. By applying metagenomic sequencing, we further identified several species of *Bifidobacterium* with high abundance in EPG, namely *B. pseudocatenulatum*, *B. longum*, and *B. bifidum*, which showed positive correlations with body weight and ADG. Probiotic effects of *Bifidobacterium* members on other animals have been reported, including improvement of host metabolism^30^, inhibition of tumor growth^31^, promotion of infant growth^32^, and relief of gut inflammation^33^. In this study, we used calves as a model to verify that probiotics can also promote the growth of young animals. Of note, some *Bifidobacterium* members originate from specific hosts^34^. Relying on the exclusive database of ruminant GIT, we identified several *Bifidobacterium* strains in calf feces, among which *Bifidobacterium longum AGR2137* has reportedly been successfully isolated only from the feces of calves^35^. These results suggested that the favorable growth phenotype of calves may be driven by hindgut-specific *Bifidobacterium*.

Metabolic liability also significantly differed between EPG and PPG. The prediction of microbial host bacteria executing metabolic functions showed that *Bifidobacterium* species were the primary microbial origins of metabolic function genes in EPG, suggesting that the difference in dominant bacteria drove the heterogeneity of corresponding metabolic functions. Specifically, processes involving degradation of exogenous carbohydrates (starch, sucrose, and cellulose) were more active in EPG. Most *Bifidobacterium* taxa tend to use monosaccharides, disaccharides, and oligosaccharides, whereas the genomes of *B. longum* and *B. pseudocatenulatum* contain various glycan-active enzymes, which are recognized for the ability to efficiently degrade plant-based carbohydrates^36,37^. Therefore, in this study, adequate daily intake of milk replacer (MR) before weaning promoted the proliferation and metabolism of *Bifidobacterium*, and the high abundance of *B. longum* and *B. pseudocatenulatum* explained the higher level of forage consumption by EPG calves. In the downstream metabolic pathways, metabolism of VFAs was also enriched by the upregulated functional genes in EPG; specifically, the processes of acetate, propanoate, and butanoate synthesis became active. VFAs were the most abundant substance in the degradation of carbohydrates by gut microbiota. As an important bridge, VFAs produced by the gut microbiota constitutes the interaction network between the microbiome and its host^38^. Butyrate has been identified as the most important energy substance for intestinal development. Up to 95% of the butyrate produced by the microbiota is absorbed and used by the colon to maintain and develop epithelial structure^39^. In addition, as a ligand, butyrate binds to histone deacetylases to regulate the expression of peroxisome proliferator-activated receptors and maintain intestinal homeostasis^40^. Propanoate produced by the microbiota can be converted to glucose through gluconeogenesis in the liver for consumption by the host^41^. Acetate is the most abundant fatty acid produced by the gut microbiota. The lack of acetate is closely related to abnormal lipid consumption and metabolism of hosts^42^. As an important precursor of VFAs, lactate is one of the main products of *Bifidobacterium*^43^. Furthermore, *Bifidobacterium* has the ability to directly convert starch into short chain fatty acid (SCFAs). In this study, we speculated that a variety of *Bifidobacterium* members in the hindgut of EPG calves were the main driving force of VFA synthesis. Similarly, *Bifidobacterium* also stimulated the synthesis of amino acids in EPG. One study demonstrated that different types of *Bifidobacterium* were responsible for a specific and diverse set of gene functions. These functions include *Bifidobacterium* bile salt hydrolase (BSH), which hydrolyzes bound bile salt into free bile acids and amino acids^44^, for which *B. longum* showed a stronger ability in amino acid synthesis and metabolism compared with other strains^45^. *Bifidobacterium* has also been shown to influence or directly participate in the synthesis of vitamins, which are indispensable active substances that promote the development of young animals and maintain their health. *B. longum* improved colitis in mice by regulating the metabolism of vitamin B6^46^. *B. brevis* produced glutathione and vitamin B6 to meet the host’s demands for antioxidants^47^. Consistently, we also observed the activation of vitamin B6 synthesis in this study.

Interestingly, compared with the enrichment of methanogens and the methanogenic pathway in PPG, methane synthesis seemed to be inhibited in EPG. Statistically, greenhouse gas emissions from the entire animal husbandry production chain account for about 15% of global man-made greenhouse gas emissions^48^. As one of the main greenhouse gases, it is of great ecological significance to effectively reduce methane emissions from animal sources. Previous studies revealed that high abundance of methanogens in the animal gut was negatively correlated with fat deposition^49^; thus, regulation of gut methanogens might be an effective way to promote animal growth^50^. Mechanistically, the inhibition of methane production essentially involves a competition for hydrogen ions between methanogens and other nutrient-metabolizing bacteria. One study reported that acetate-producing bacteria, which are more attracted to hydrogen (H_2_), promote the production of volatile acids rather than methane by the gut microbiota^51^. Hence, we reasoned that the more abundant *Bifidobacterium* in EPG compared with PPG used more H_2_ to produce lactose and acetate. The resulting construction of a nutrient-producing microbial community instead of a methane-producing one led *Bifidobacterium* to occupy a more dominant niche in EPG, thus increasing the hindgut energy harvested for calf growth.

In this study, it was a limitation that the present study did not weigh the male calves due to the feeding and management protocol of the commercial farm, and it would be better if the future study could include growth phenotype from both male and female animals. Additionally, although in this experiment, we proved the beneficial effect of *Bifidobacterium*-enriched fecal microbiome on the host, due to the complexity and diversity of fecal microbial community, we could not remove potential interferences of other bacteria, especially pathogens, on the host, which posed a challenge for us to further explore the probiotic mechanism of *Bifidobacterium* and screen related marker metabolites. Therefore, we anticipated that the functional identification and verification of single *Bifidobacterium* strain and related metabolites might be a future research direction that could help guide the development of more accurate, efficient growth-promoting probiotics for animal development.

This study investigated the invasion and dynamic changes of core bacterial taxa in the early life of calves. Enterotype analysis based on large samples generated four calf enterotypes, two of which (dominated by *Bifidobacterium* and *Oscillospiraceae_UCG-005*) were identified as representative enterotypes. The frequency of conversion between enterotypes shifted from variable to stable with age. Functional profiling revealed distinct, enterotype-specific characteristics and transition trends. Additionally, we observed a close relationship between phenotype and enterotype, with the *Bifidobacterium*-dominant enterotype showing better growth performance. The *Bifidobacterium* members improved calf growth by promoting the use of carbohydrates, activating the synthesis of VFAs, amino acids, and vitamin B6, and inhibiting methane production in the hindgut. The FMT in mice further validated the beneficial effect of the *Bifidobacterium*-dominant microbiota on animal growth and hindgut development.

## Methods

### Animals and experimental design

The animal experiment was conducted at the Gansu Tianmu Farm (Jin Chang, Gansu Province, China). A large cohort of 408 Holstein dairy calves (150 female and 258 male) were enrolled in this study and they were all fed followed by the dietary regime of farm from birth to 70 days of age (Supplementary Table 2). Specifically, the calves were fed milk and milk replacer (MR) twice a day (08:00 and 16:00) according to the Table S1. The calves had unrestricted access to water, concentration, and forage from birth to 70 days of age. The nutritional composition of each feed was shown in the Supplementary Table 3. During the period, considering feeding and management protocol of the commercial farm, we only tracked and recorded the growth performance and health of 150 female calves from their birth to 70 days of age. The feed intake (milk, concentration, and forage), diarrhea condition, and antibiotic treatment of each female calf were recorded every day. Their body weights were measured utilizing a calf weighing machine at specific time points: 0, 14, 28, 42, 56, and 70 days of age. We also collected related information about the cow corresponding to each calf, including BCS, BW, morbidity, gestation day, parity, age, and antibiotic treatment.

### Collection of calf samples

Fecal samples (*n* = 1045) were collected from each calf at their corresponding 0, 14, 28, 42, 56, or 70 days of age. The sampling time points of fecal samples at 14, 28, 42, 56, and 70 days of age were all at 06:00 before the morning feeding of calves. Notably, the meconium samples from calves on day 0 were collected within half an hour of birth before colostrum administration. All fecal samples were collected using sterilized gloves and stored in the 5 mL sterilized frozen storage tubes. Blood samples were collected from female calves at 06:00 at 70 days of age by jugular vein sampling and stored in 10 mL evacuated tubes. The blood samples were further centrifuged at 3000 × *g* for 10 min at 4 °C to collect the serum in the 5 mL sterile frozen storage tubes. Both fecal and serum samples were snap frozen in liquid nitrogen and then stored in −80 °C refrigerator for the subsequent analysis.

### DNA extraction, PCR amplification, and data processing

A total of 1,045 fecal samples of calves were collected for 16S sequencing. A Dneasy PowerLyzer PowerSoil Kit (Qiagen, Inc., Germantown, MD, USA) was used to extract microbial DNA from fecal samples. The quality of total DNA preparations was checked using a Thermo NanoDrop 2000 UV microphotometer and 1% agarose gel electrophoresis. The V3-V4 region of the bacterial 16S rRNA gene was amplified using the primer pair 338 F (5′-ACTCCTACGG GAGGCAGCAG-3′) and 806 R (5′-GGACTACHVGGGTWTCTAAT-3′) in an ABI GeneAmp® 9700 PCR thermocycler (ABI, CA, USA). Of note, we also set up negative and positive controls in the PCR amplification process to ensure that the samples did not receive reagents or environmental contamination (Supplementary Fig. 12). The PCR products were extracted from 2% agarose gels and purified using the AxyPrep DNA Gel Extraction Kit (Axygen Biosciences, Union City, CA, USA), in accordance with the manufacturer’s instructions, and quantified using Quantus™ Fluorometer (Promega, Madison, Wisconsin, USA). Amplicon libraries were sequenced using an Illumina Miseq PE250 platform (Illumina, San Diego, CA, USA).

Raw FASTQ files were de-multiplexed using an in-house Perl script, then quality-filtered by fastp version 0.19.6 and merged by FLASH version 1.2.11^52^. DADA2 was chosen to de-noise the optimized sequences. Taxonomic assignment of amplicon sequence variants was then performed using the naive Bayes consensus taxonomy classifier implemented in Qiime2 and the SILVA 16S rRNA database (v138), and then adjusted for the rRNA operon copy number estimated using data from the rrnDB database^53^.

### Metagenomic sequencing and processing

A total of 20 fecal samples (10 replicates per group) were selected randomly from the calves in the different enterotype groups of 70 days of age to perform the metagenomic shotgun sequencing. Moreover, we calculated the statistical power using G*Power software (version 3.1.9.7; https://g-power.apponic.com) to obtain power (1 − β prob) of 0.80 and α err prob error of 0.05. The effect size for microbial difference was determined referred by the previous research^3^ (value = 1.678). The result of calculation showed the minimum sample size was 10. Therefore, the sample size of 20 fecal samples was sufficient to the microbial analysis.

Extracted DNA was fragmented to an average size of about 400 bp by a Covaris M220 ultrasonicator (Gene Company Limited, Shanghai, China) for construction of a paired-end library using NEXTFLEX Rapid DNA-Seq (Bioo Scientific, Austin, TX, USA). Paired-end sequencing was performed on an Illumina Novaseq 6000 (Illumina Inc.) using a NovaSeq 6000 S4 Reagent Kit, in accordance with the manufacturer’s instructions at www.illumina.com. The raw sequencing reads were trimmed of adapters, and low-quality reads (length < 50 bp; quality value < 20; or presence of N bases) were removed by fastp (https://github.com/OpenGene/fastp, version 0.20.0). Reads were aligned to the *Bos taurus* genome and any hits associated with the reads or their mated reads were removed. The quality-filtered data were assembled using MEGAHIT (https://github.com/voutcn/megahit, version 1.1.2). Contigs with a length ≥ 300 bp were selected as the final assembling result. Open reading frames (ORFs) from each assembled contig were predicted using Prodigal (https://github.com/hyattpd/Prodigal, version2.6.3), and ORFs ≥ 100 bp in length were retrieved. A non-redundant gene catalog with 90% sequence identity and 90% coverage was constructed using CD-HIT (http://weizhongli-lab.org/cd-hit/, version 4.7). Gene abundance for a specific sample was estimated with 95% identity by SOAPaligner (https://github.com/ShujiaHuang/SOAPaligner, version soap2.21release). The best-hit taxonomy of non-redundant genes was obtained by aligning them against the NCBI NR database using DIAMOND (http://ab.inf.uni-tuebingen.de/software/diamond/, version 2.0.11) with an e-value cutoff of 1e-5. The functional annotation of non-redundant genes at KEGG and CAZy was similarly obtained^54^.

### Metabolite extraction and quality control

Similarly, the fecal and serum samples from the same 20 calves were also used to detect the metabolic profiles. Each 50-mg fecal and serum sample was added to a 2-mL centrifuge tube, followed by a 6-mm diameter grinding bead. The samples were ground by a Wonbio-96c frozen tissue grinder (Shanghai Wanbo Biotechnology Co., Ltd., Shanghai, China) for 6 min (−10 °C, 50 Hz) and extracted for 30 min (5 °C, 40 kHz) using low-temperature ultrasonication. After leaving the samples at (−20 °C for 30 min) and centrifuging for 15 min (4 °C, 13,000 × *g*), the supernatant was collected for subsequent analysis. Quality control samples comprising a mixture of equal volumes of all samples were prepared to monitor the stability of the analysis.

### Metabolomics data analysis

The liquid chromatography-tandem mass spectrometry analysis of samples was conducted on a Thermo UHPLC-Q Exactive HF-X system equipped with an ACQUITY HSS T3 column (100 mm length × 2.1 mm inner diameter; 1.8 μm particle size; Waters Corp., Milford, Massachusetts, USA). Under both positive and negative ion modes, a TripleTOF 5600 Plus high-resolution tandem mass spectrometer (SCIEX, Warrington, UK) was used to identify metabolites eluted from the column. Acquired data were exported into the mzXML format using XCMS software^55^. Analyses of traceability and enrichment of metabolites were performed using MetOrigin (http://metorigin.met-bioinformatics.cn/app/metorigin)^17^. At the MetOrigin online server, the Simple MetOrigin Analysis (SMOA) mode, which requires a list of metabolites with KEGG or Human Metabolome Database IDs, was chosen for our data. The SMOA mode allows identification of the origins of metabolites based on seven well-known metabolite databases. After loading the dataset, metabolic pathway enrichment analysis analysis was carried out. A bar plot was created to summarize the total number of metabolites from the host, microbiota, both host and microbiota, and other sources.

### FMT in mice

The operation of fecal microbial suspension was performed in the ultra-clean worktable, and the related equipment and consumables were also sterilized under high temperature and high pressure. The frozen fecal samples of the EPG and PPG stored at −80 °C were thawed in a constant temperature water bath at 37.5 °C (about 10 min). Next, a 1 g fecal sample for each calf in the two groups (10 g per group) was homogenized and diluted in 90 ml PBS buffer containing 0.2 g/l Na2S and 0.5 g/l cysteine. We used sterile screens with 200 mesh, 400 mesh, and 800 mesh in sequence to filter out insoluble particles and impurities and centrifuged the filtrate at 600 × *g* for 5 min, and removed the supernatant fluid into 10 mL centrifuge tubes. Then a drop of suspension absorbed by a straw was placed on the edge of the cover slide in the center of the XB-K-25 blood cell count board, keeping it to slowly seep into the counting room. According to the established counting method and calculation formula, the concentration of suspension was determined (over than 10^8^ CFU/mL in the both group). Finally, we added 10% sterile glycerol to the fecal microbial suspension, sealed the centrifuge tubes with sealing films and stored them in a refrigerator at −80 °C for the following FMT.

A total of 24, 3-week-old female specific pathogen free (SPF) C57BL/6 J mice (Sibeifu Biotechnology Co., Ltd., Beijing, China) were enrolled in the FMT experiment and underwent a 7-day acclimatization stage before treatment. All the mice were kept in the SPF animal barrier facilities and fed with a normal diet and purified water. The feeding conditions were room temperature (23.0 ± 2.0 °C), relative humidity of 50–60%, and 12 h of light every day. Next, the mice were treated with antibiotics cocktail (ampicillin 1 g/l, neomycin 1 g/l, metronidazole 1 g/l, vancomycin 0.5 g/l, diluted in ultra-pure water) for 7 days to deplete the gut microbiome. In detail, the mice had unrestricted access to antibiotics cocktail water and also administered an additional 200 μL/day of antibiotics cocktail. After 7 days, the mice experienced 4-day wash-out period to eliminate the residual antibiotics before FMT. Subsequently, these mice were randomly divided into 3 groups (*n* = 8; 4 mice/cage; CON, EPG and PPG), which were colonized by oral gavage with 200 μL/d of PBS, fecal microbial suspension from EPG calves or PPG calves for 2 weeks. During the period, we measured the weight of the mice every day. At the end of 14-day FMT experiment, the mice were placed in a sealed euthanasia box connected to the carbon dioxide (CO_2_) input pipe, then the input valve was opened and CO_2_ was infused into the box at a rate of 10–30% of the volume of the euthanasia box per minute. When the mice were motionless, not breathing, and their pupils were dilated. We closed the valve and observed the mice for another 2–3 min to determine their death. The colonic tissues of mice were collected to make the paraffin sections for subsequent histological observation. The colonic contents were also collected in the 5 mL sterilized frozen storage tubes and stored in the −80 °C refrigerator for the next microbial analysis.

### Histomorphologic examinations

Colonic tissue samples were dehydrated in a series of ethanol solutions, embedded in paraffin sections, and cut into 6-μM sections. The sections were stained with hematoxylin, and the colonic structure was observed under an Olympus BX-51 light microscope (Olympus Corporation, Tokyo, Japan) at 10× magnification. The microscopic evaluations were conducted blindly by an experienced pathologist.

### Statistics

Alpha diversity (Shannon and Chao1 indexes) in the groups was compared using the Kruskal–Wallis test and a post-hoc Dunn’s multiple comparison with a Bonferroni adjustment. Beta diversity based on Bray-Curtis distances was calculated and tested using an analysis of similarity (ANOSIM). The outputs of diversity were visualized using the ggplot2 R package (version 3.6.0). LEfSe, an analytical tool for discovering and interpreting biomarkers of high-dimensional data, was used to identify the signature microbiota. *p* < 0.05 and LDA score > 2 were used as a criterion for judging the significant effect size.

The enterotype analysis was performed using Jensen-Shannon divergence distance and PAM clustering. The optimal number of clusters was calculated using the CH index.

The random forest regression model was used to identify age-related bacteria in calf feces. The random Forest R package (version 4.6-14, available at https://cran.r-project.org/web/packages/randomForest/) was used, with the parameters of ‘importance’ and “proximity” set as “True”, and “ntree” set to 10,000 trees.

Spearman’s analysis was performed to calculate correlations between bacteria in different clusters using the psych R package, and the related interaction networks were visualized using Gephi (https://gephi.org/). Only significant coefficients (*P* < 0.05, |r| > 0.5) are shown in the networks. The degree of nodes was applied to represent the network sparsity; the lower the degree, the sparser the network is. The natural connectivity of a complex network was used to reveal the robustness of the network. Line plots were illustrated using the ggplot2 R package.

### Ethics approval

The animal experiment in this study was performed in accordance with the Regulation of the Administration of Laboratory Animals (2017 Revision) promulgated by Decree No. 676 of the State Council, China. The animal care protocol was approved by the Animal Care and Use Committee of China Agricultural University (Protocol Number: AW10803202-3-2).

### Reporting summary

Further information on research design is available in the Nature Research Reporting Summary linked to this article.

### Supplementary information

supplementary

Reporting summary

## Data Availability

The accession for the 16S sequencing and metagenomics data in this study is NCBI Sequence Read Archive: # PRJNA1052964. The metabolomic data has been deposited in National Genomics Data Center (https://ngdc.cncb.ac.cn) under the BioProject PRJCA027522.
