# Supplementary material for: The Bifidobacterium-dominated fecal microbiome in dairy calves shapes the characteristic growth phenotype of host
Source: NPJ Biofilms Microbiomes. 2024 Jul 21;10:59. doi: 10.1038/s41522-024-00534-4 (PMC11271470; doi:10.1038/s41522-024-00534-4)
Supplement: Supplementary file 1 — supplementary [file 41522_2024_534_MOESM1_ESM.pdf]

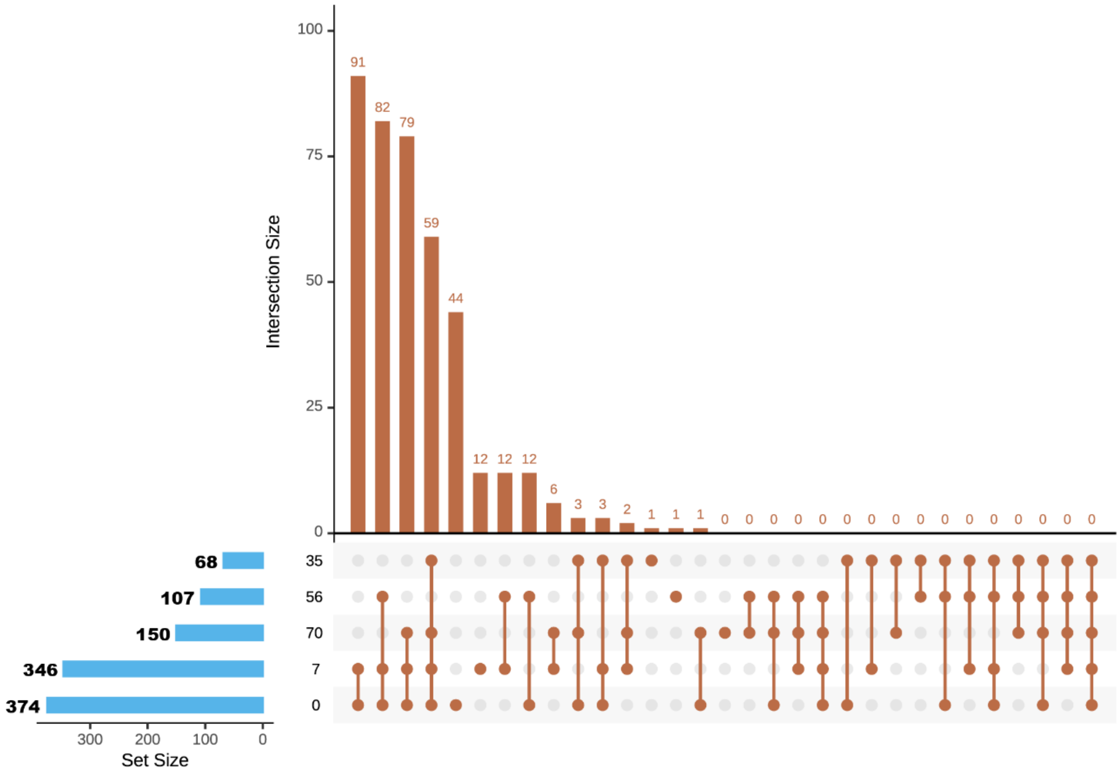

Supplementary Fig. 1. Temporal prevalence of 1,045 fecal samples of calves used in this study.

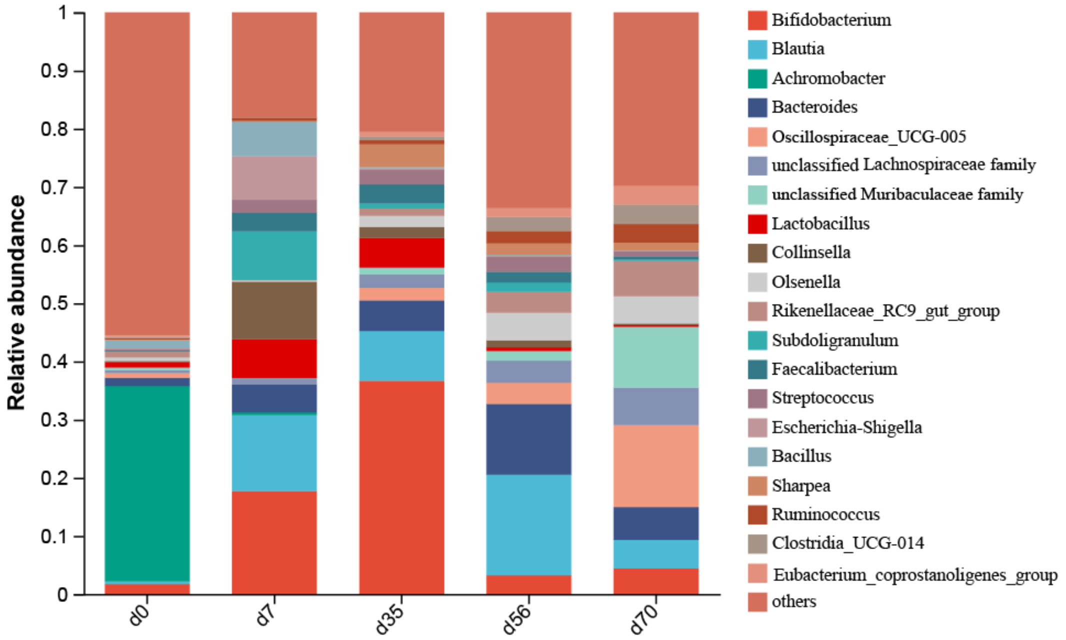

Supplementary Fig. 2. Mean relative abundance of top 20 bacteria in abundance at the genus level across the samples in the different age groups. The abundance of others = the sum of the abundance of bacteria outside the top 20.

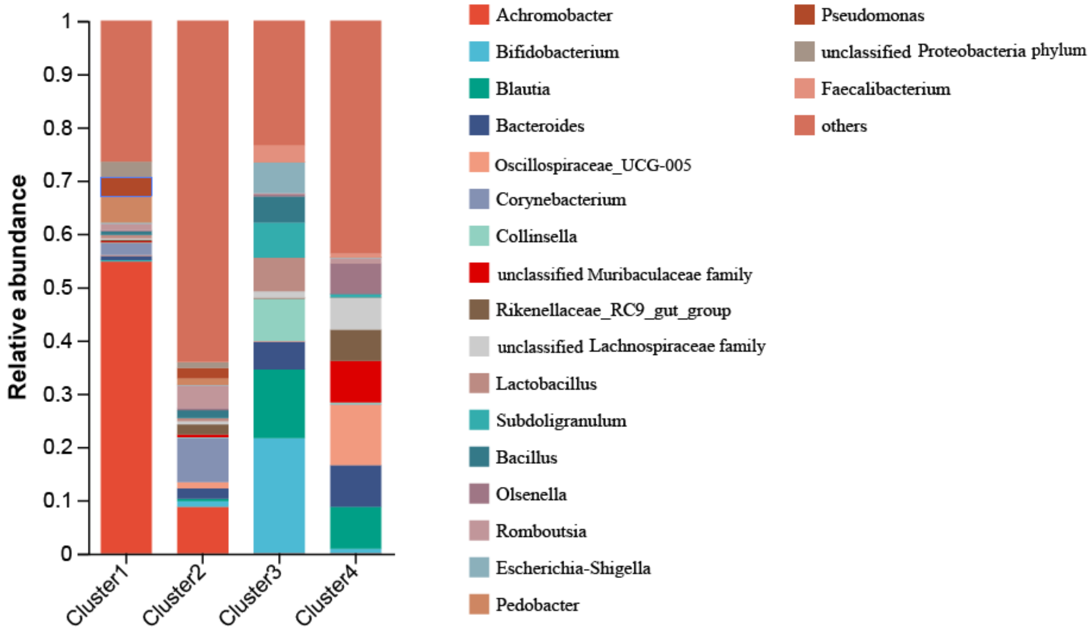

Supplementary Fig. 3. Mean relative abundance of top 20 bacteria in abundance at the genus level across the samples in the different enterotype groups. The abundance of others = the sum of the abundance of bacteria outside the top 20.

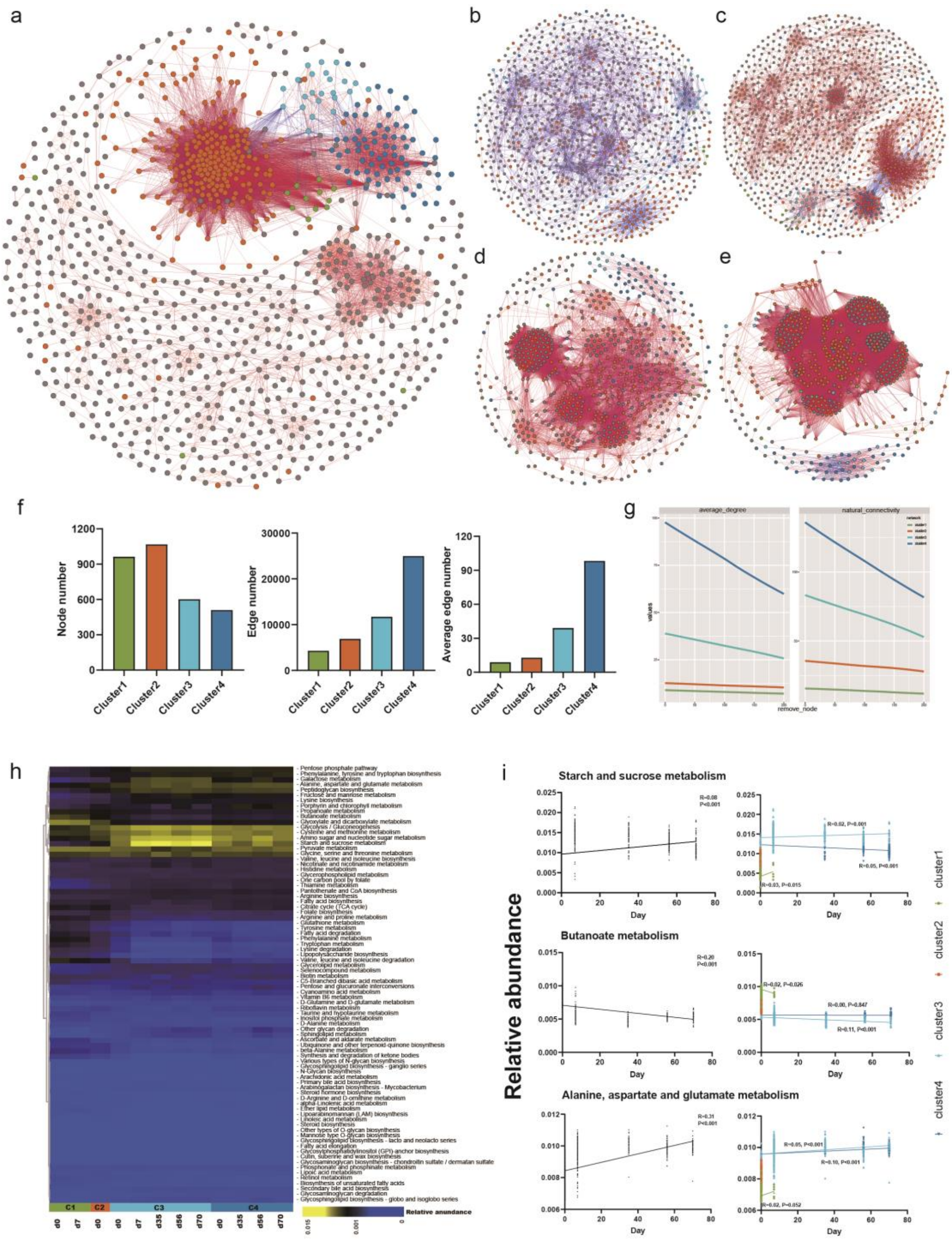

Supplementary Fig. 4. The microbial interaction network and functional profile in the different enterotypes. **a** Co-occurrence interaction network of the gut microbiota during the whole period. The microbial network in the cluster 1 (**b**), cluster 2 (**c**), cluster 3 (**d**) and cluster 4 (**e**). The signature genera (identified by LEfSe) were distinguished by different colors in their name. Green: The signature bacteria in the cluster 1; Orange: The signature bacteria in the cluster 2; Light blue: The signature bacteria in the cluster 3; Blue: The signature bacteria in the cluster 4; **f** The number of node and edge in the enterotypes networks. **g** The average degree and natural connectively of networks in the four enterotypes networks. **h** The heatmap of metabolic pathways of four enterotypes from 0 to 70 days of age. **i** The change trend

of pathway of “Starch and sucrose metabolism”, “Butanoate metabolism” and “Alanine, aspartate and glutamate metabolism” in the different enterotypes with age.

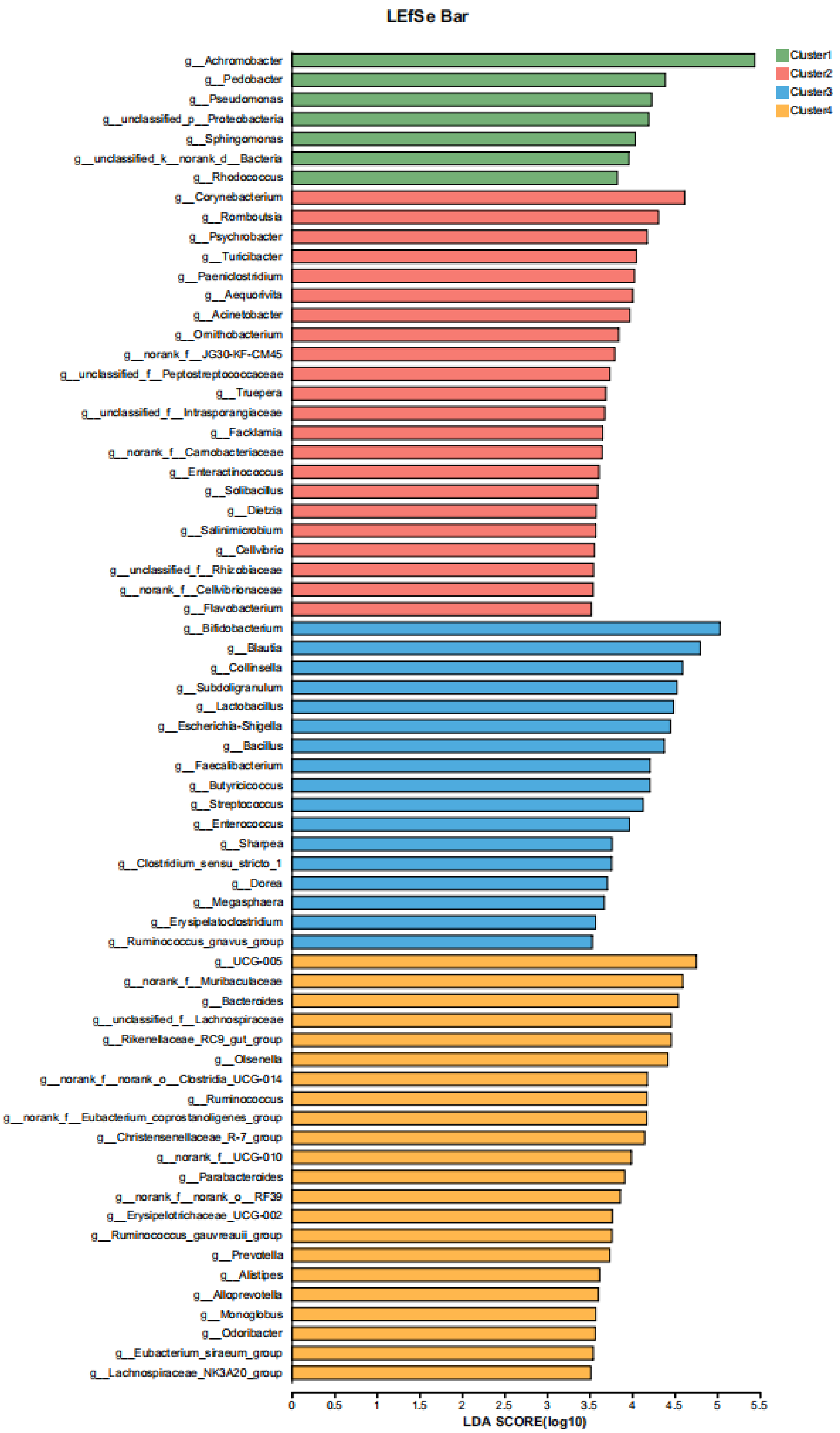

Supplementary Fig. 5. The enterotype-associated genera identified by LEfSe

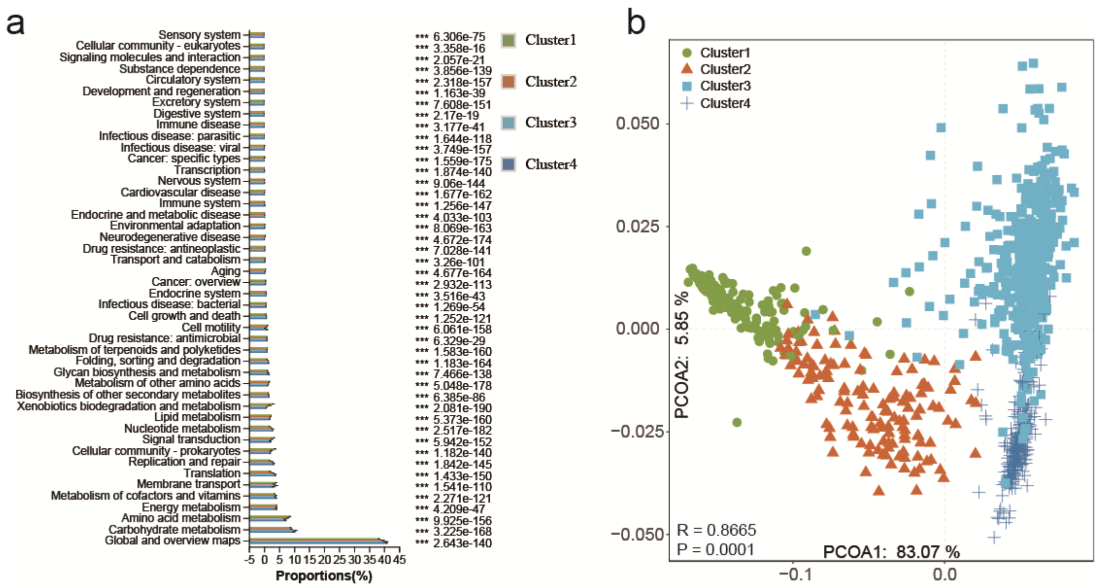

Supplementary Fig. 6. The functional profile in the four enterotypes. **a** The significant difference of KEGG pathways at the level 2 in the four enterotypes. The significance was assessed using Kruskal-Wallis test. **b** The β-diversity of functional genes of four enterotypes. The significance was assessed using ANOSIM.

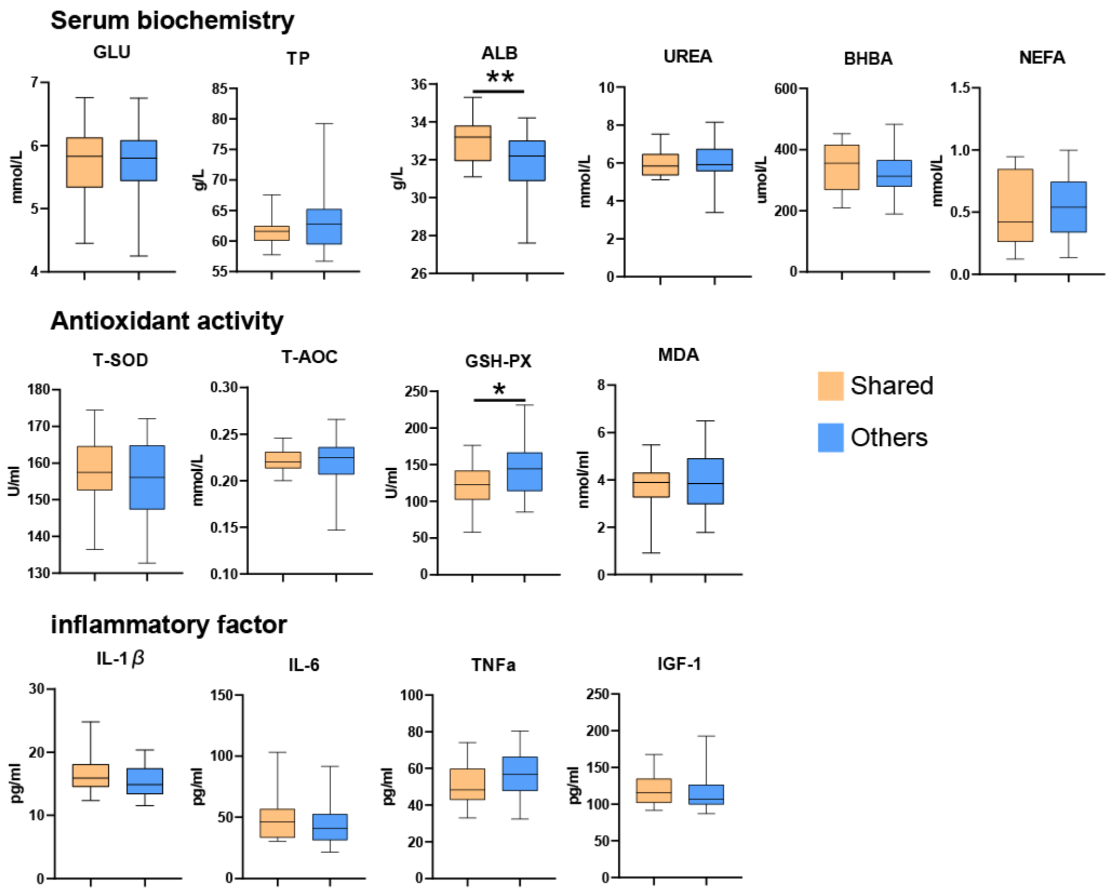

Supplementary Fig. 7. The comparison of serum indicators in the shared and others groups. Shared group: the calves in cluster 3/PAM 1; Others group: all other 70-day-old calves. The five horizontal lines in the box plots represented the upper limit, the upper quartile, the median, the lower quartile and the lower limit. The significance between groups was assessed using one-way ANOVA. Only the significant differences were marked (*$p < 0.05$, **$0.01 < p < 0.05$, ***$p < 0.001$).

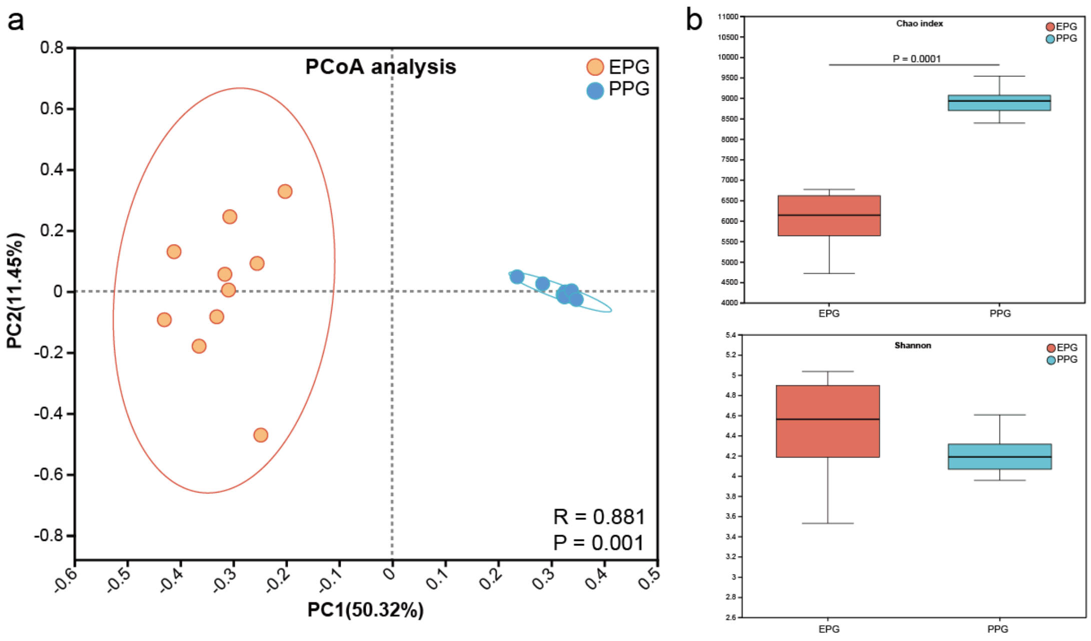

Supplementary Fig. 8. The diversity of gut microbiome in the EPG and PPG. **A** The principal coordinate analysis (PCoA) of gut microbiome in the EPG and PPG. The five horizontal lines in the box plots represented the upper limit, the upper quartile, the median, the lower quartile and the lower limit.The significance was assessed using ANOSIM. **B** The Chao1 and Shannon index between the EPG and PPG. The five horizontal lines in the box plots represented the upper limit, the upper quartile, the median, the lower quartile and the lower limit. The significance between groups was assessed using Wilcoxon rank-sum test.

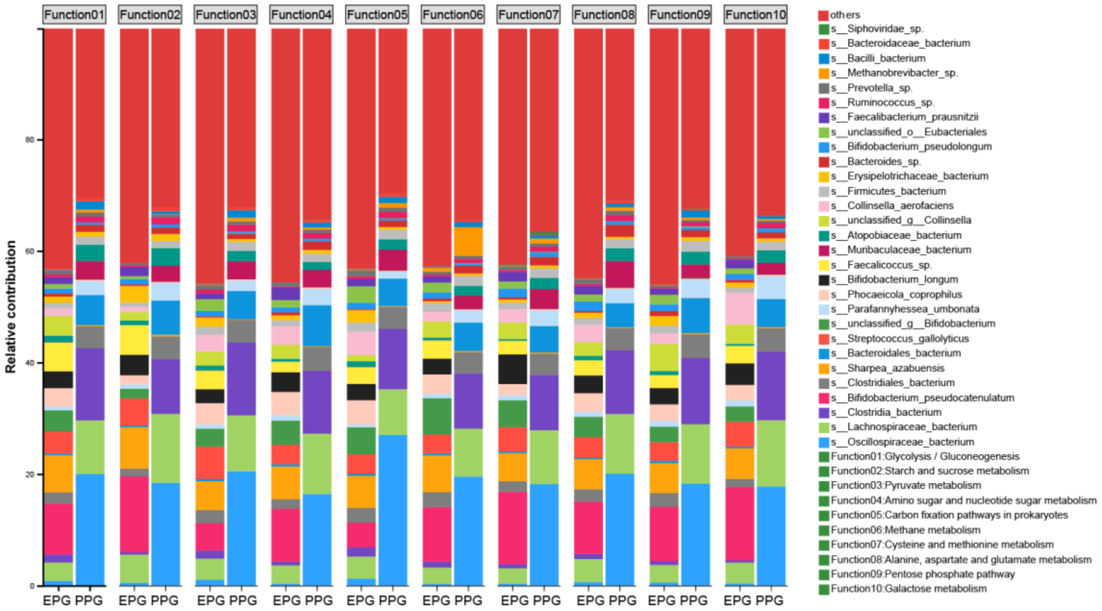

Supplementary Fig. 9. The prediction of microbial host in the top 10 metabolic pathways (KEGG level3) at the species level between the EPG and PPG.

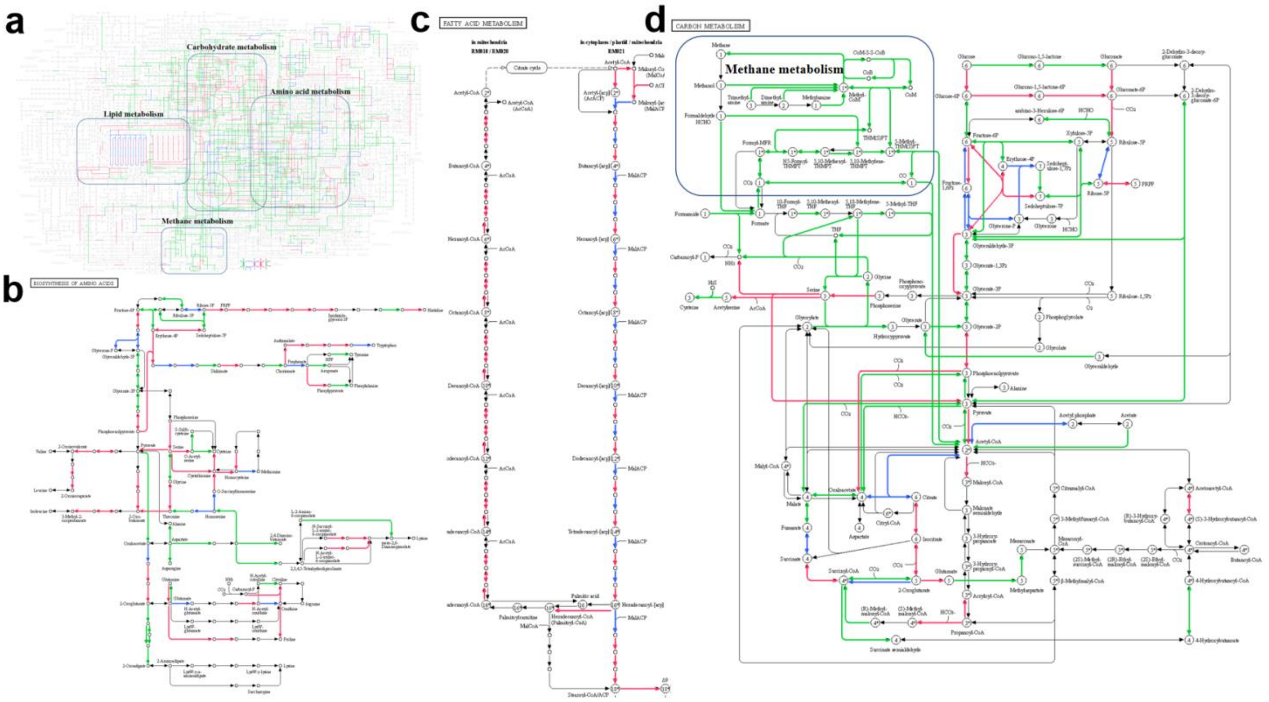

Supplementary Fig. 10. The functional enrichment of differential expressed genes (DEG). **a** The global metabolism. **b** Biosynthesis of amino acid. **c** Fatty acid metabolism. **d** Carbon metabolism.

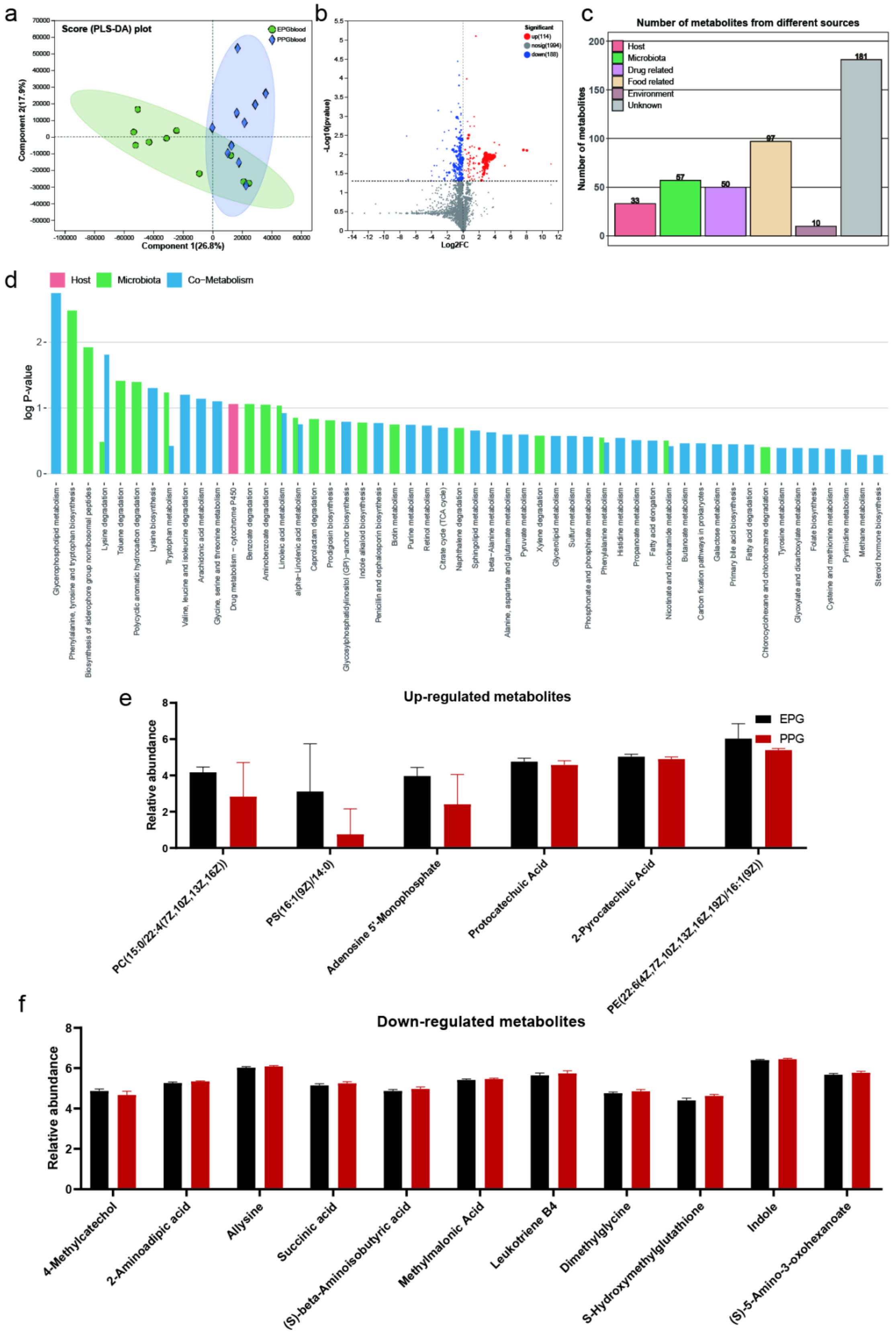

Supplementary Fig. 11. The blood metabolic profile of the EPG and PPG. **a** Partial

least squares-discriminant analysis (PLS-DA) of the blood metabolome between the EPG and PPG calves. **b** Volcano map of metabolites identified by the blood metabolome. **c** The identification of fecal metabolites from different sources. **d** Metabolic pathway enrichment analysis according to different categories of metabolites belonging to the different sources. **e** The up-regulated metabolites enriched in the metabolic pathways. **f** The down-regulated metabolites enriched in the metabolic pathways. The error pounds in the bar charts were represented by mean +/- SD.

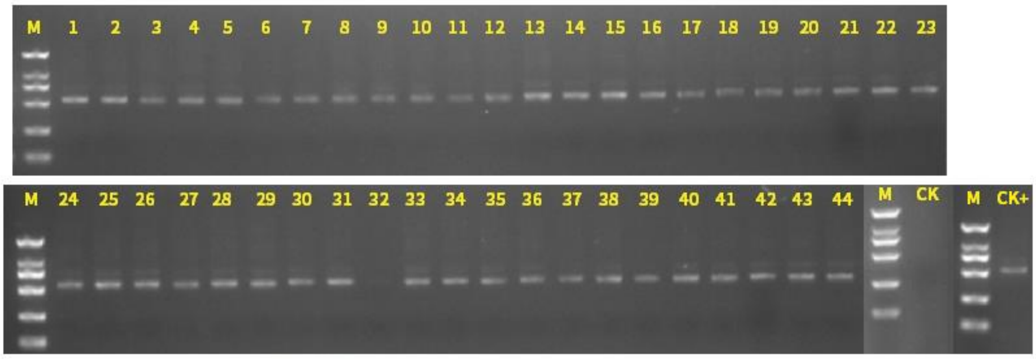

Supplementary Fig. 12. PCR amplification electropherogram of fecal microbial samples. 1-44: fecal samples; CK: negative control (ultra-pure water); CK+: positive control (microbial community standard).

Supplementary Table 1. Analysis of similarity (ANOSIM) test for microbiota dissimilarities at different ages of calves based on the Bray-Curtis distance

Items	Group1	Group2	*R*	*P -value*
Bray-Curtis	Day1	Day7	0.89	< 0.001
	Day1	Day35	0.85	< 0.001
	Day1	Day56	0.79	< 0.001
	Day1	Day70	0.80	< 0.001
	Day7	Day35	0.29	< 0.001
	Day7	Day56	0.59	< 0.001
	Day7	Day70	0.82	< 0.001
	Day35	Day56	0.60	< 0.001
	Day35	Day70	0.64	< 0.001
	Day56	Day70	0.49	< 0.001

Supplementary Table 2. The liquid feed regime of calves

Liquid feed regime	Day	Volume/L
Colostrum	1h after birth	4
Wasted Milk	1~2	5
Wasted Milk	3~7d	6
Wasted Milk	8~14	8
Wasted Milk	15-22	10
Wasted Milk +Milk Replacer	22-25	10
Milk Replacer	26~48	10
Milk Replacer	49~51	8
Milk Replacer	52~54	6
Milk Replacer	55~56	4
Milk Replacer	57~70	0

Supplementary Table 3. Nutritional components of calf feed

Items	Starter	Forage	Wasted Milk	Milk Replacer
Dry Matter（%）	88.4	87.6	14.35	96
Crude Protein（%）	23.98	7.35	3.75	22
Ether Extract（%）	2.51	2.26	4.36	16
Lactose（%）	-	-	5.52	46
Neutral Detergent Fiber（%）	20.4	50.24	-	-
Acid Detergent Fiber（%）	9.65	29.95	-	-
Ash（%）	7.43	13.4	0.79	-
Ca（%）	-	-	-	0.9
P（%）	-	-	-	0.75
